# A Comprehensive Review of Azelaic Acid Pharmacological Properties, Clinical Applications, and Innovative Topical Formulations

**DOI:** 10.3390/ph18091273

**Published:** 2025-08-26

**Authors:** Andreea-Georgiana Petrovici, Mariachiara Spennato, Ioan Bîtcan, Francisc Péter, Livius Cotarcă, Anamaria Todea, Valentin Laurențiu Ordodi

**Affiliations:** 1 Biocatalysis and Green Chemistry Group, Faculty Chemical Engineering, Biotechnologies and Environmental Protection, University Politehnica Timisoara, Vasile Pârvan 6, 300223 Timisoara, Romania; andreea.petrovici@student.upt.ro (A.-G.P.); ioan.bitcan@upt.ro (I.B.); francisc.peter@upt.ro (F.P.); valentin.ordodi@upt.ro (V.L.O.); 2 Department of Civil, Chemical, Environmental and Materials Engineering, Alma Mater Studiorum University of Bologna, 40126 Bologna, Italy; mariachiara.spennato@unibo.it; 3 Renewable Energy Research Institute-ICER, University Politehnica Timisoara, Gavril Musicescu 138, 300501 Timisoara, Romania; 4 LC Consulting SAS, Via Martignacco 117, 33100 Udine, Italy; livius.cotarca@inwind.it

**Keywords:** azelaic acid, topical drug delivery, nanocarriers, liposomes, solid lipid nanoparticles, nanocrystals, microemulsions, deep eutectic solvents, controlled release, formulation development

## Abstract

Azelaic acid (AzA), a saturated dicarboxylic acid, is indicated for the treatment of acne vulgaris, rosacea, melasma, and post-inflammatory hyperpigmentation. Its antimicrobial, anti-inflammatory, and antimelanogenic properties support its use; however, its poor aqueous solubility and limited skin permeability constrain its optimal topical delivery. This review summarizes clinical evidence and advances in formulations—including conventional vehicles, polymeric/lipid nanocarriers, and deep eutectic solvent (DES) systems—to promote more effective and well-tolerated use. Across indications, 15–20% azelaic acid (AzA) formulations produced clinically meaningful improvements with mild, transient local irritation. For acne vulgaris, reductions in inflammatory and noninflammatory lesions were comparable to those of topical retinoids/adapalene, and tolerability was superior in some studies. For rosacea, the 15% gel formulation was comparable to metronidazole in reducing papules, pustules, and erythema while maintaining negligible systemic exposure. In melasma and other dyschromias, 20% cream demonstrated efficacy similar to hydroquinone, exhibiting a favorable safety profile. Advanced delivery systems, including liposomes, niosomes/ethosomes, nanostructured lipid carriers, microemulsions, nanosponges, and DES platforms, increased AzA solubilization, cutaneous deposition, and stability. This enabled dose-sparing strategies and improved adherence. Data on AzA cocrystals and ionic salts suggest additional control over release and irritation. AzA remains a versatile and well-tolerated dermatologic agent whose performance is strongly vehicle-dependent. Rational selection and engineering of carriers, particularly DES-integrated polymeric and lipid systems, can mitigate solubility and permeability limitations, enhance skin targeting, and reduce irritation in the treatment of acne and rosacea.

## 1. Introduction

### 1.1. Structure, Physicochemical Properties, and Natural Occurrence of Azelaic Acid

**Figure 1:**
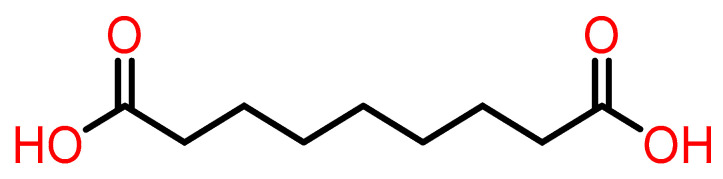
Chemical structure of azelaic acid.

Azelaic acid (AzA) is a saturated dicarboxylic acid with the molecular formula C_9_H_16_O_4_ and a molecular weight of 188.22 g/mol [1,2]. Its structure (Figure 1) comprises a linear nine-carbon chain terminated by two carboxylic acid groups (-COOH), classifying it as an aliphatic dicarboxylic acid [2]. The IUPAC name for azelaic acid is nonanedioic acid, and its CAS number is 123-99-9 [2].

Physically, it is a white, odorless crystalline powder with a melting point between 106.5 °C to 109 °C. It is sparingly soluble in water (~2.4 g/L at 20 °C) but highly soluble in ethanol and other polar solvents—important for topical formulation [2].

It has two dissociation constants (pKa 4.55 and 5.50), reflecting the deprotonation of its COOH groups. Its log P is ~1.22, indicating moderate lipophilicity that aids skin permeation [2].

AzA occurs naturally in grains such as barley and rye, and is a metabolic byproduct of *Malassezia furfur*, a yeast found on human skin [3]. It is synthesized in small amounts in the human body from fatty acids by omega-oxidation [4]. Its discovery helped spark interest in dicarboxylic acids for dermatology [5]. The natural occurrence of AzA in plants was investigated mostly in connection with its proved phytotoxic effect and consequently the potential utilization as a bioherbicide in weed control. A study of Ma et al. [6] revealed that AzA contained in the aqueous extracts of leaves and roots of *Jathropa curcas* inhibited the growth of neighboring crops. The highest amount of 0.94% AzA was found in the distilled water extract of leaves (2 g of dried plant material soaked for 7 days in 100 mL distilled water) and the authors concluded that AzA is a key compound involved in the defense mechanism of the plant. The possibility of replacing the synthetic product with plant matrices containing AzA was recently investigated by Spaggiari et al., using whole grain and whole-grain flour extracts, respectively, of wheat (*Triticum durum*). This green pathway to obtain AzA from vegetable sources resulted in moderate extraction yields, 0.96% and 3.4% (*w*/*v*) being the highest values obtained by water extraction and with the Naviglio^®^ extraction system, respectively, below the concentrations used for pharmaceutical and cosmetic purposes [7]. Although such extracts have the advantage of easy accessibility and relatively low cost, for now they cannot be considered a competitive alternative of synthetic AzA for topical applications.

AzA is an active pharmaceutical ingredient (API). FDA Orange Book of Approved Drug Products with Therapeutic Equivalence Evaluations shows a consistent list of commercially approved products formulated as aerosols, foams, cream, and gel, respectively, with seven dossier applicant holders reporting AzA concentration of 15% and 20% [8]. Similar formulation for the skin diseases treatment have been approved by EMA and are found on the EU pharma market [9].

### 1.2. Synthesis and Industrial Production: Sustainable and Conventional Routes for Azelaic Acid Production

In addition to its natural occurrence, AzA can be synthesized through various industrial and biocatalytic pathways (summarized in Table 1). The conventional industrial method involves ozonolysis of oleic acid, yielding AzA and pelargonic acid as co-products. However, this process has significant drawbacks, including high energy demand and safety concerns related to ozone handling.

To address these limitations, several greener and more sustainable methods have been developed. An industrial-scale process implemented by Matrica S.p.A. (a joint venture between Versalis and Novamont) in Porto Torres (Italy) utilizes hydrogen peroxide to oxidize vegetable oil-derived oleic acid, achieving production capacities of 25,000–30,000 tons/year [10]. This method follows a three-step protocol: oxidation of the olefinic bond to vicinal diols, further oxidation to carboxylic acids, and hydrolysis of triglyceride intermediates—reaching yields up to 80%. The product has been designed to be employed as raw material for the special polyamides industry. However, it needs advanced purification to be used as it is in the polymer industry. Moreover, the technology above results in a product out of the specification as pharma grade AzA.

A convergent synthesis and process for the open-chain C-9 compounds, valuable monomers for preparation of polyamides with specific properties, has also been reported [13,14]. Starting from relatively inexpensive raw materials, for example, cyclohexanone and activated C-3 olefins, the method provides polymer grade ω-functionalized nonanoic acids, including pharma grade AzA. Alternative catalytic and chemo-enzymatic routes have also been explored. Some use in situ-generated performic acid, and others apply transition metal catalysts like tungstic or molybdic acids, with yields ranging from 70% to 95%. Notably, rare earth triflates and gold-supported catalysts have enabled selective oxidation under aqueous, mild conditions [11].

Biocatalytic routes employ enzymes such as lipases for the epoxidation of oleic acid, followed by oxidative cleavage with H_2_O_2_ or atmospheric oxygen. Yields vary—typically between 40% and 70%—but these approaches offer advantages in selectivity and environmental compatibility. One method using a lipase/TEMPO/Fe(NO_3_)_3_ system achieved 44% yield without chromatographic purification. Fermentative strategies using Candida tropicalis have also been patented, offering a PA-free route with yields around 67%, although with longer processing times [12].

Since the 1970s, research has highlighted its antimicrobial, anti-inflammatory, and anti-keratinizing actions, leading to its approval as a topical agent [15].

Clinically, AzA is used to treat acne vulgaris, rosacea, melasma, and post-inflammatory hyperpigmentation. Randomized controlled trials have validated its effectiveness—particularly the 20% cream and 15% gel formulations—showing reductions in inflammatory lesions and erythema, with good tolerability in sensitive skin [16,17,18,19]. These formulations have proven comparable, and sometimes superior, to metronidazole and adapalene [17,18].

However, formulation remains a challenge due to low water solubility and moderate lipophilicity of AzA, which limits its topical bioavailability. To overcome these limitations, new delivery systems—including liposomes, nanocarriers, and deep eutectic solvents (DESs)—have been developed to improve skin penetration, drug stability, and patient compliance [19,20,21,22,23,24].

### 1.3. Global Approvals, Usage Patterns, and Market Trends for Azelaic Acid Products

Its global regulatory status underscores its clinical relevance. In the US, the FDA has approved 15% gel for rosacea and 20% cream for acne. In Europe, those formulations are approved in multiple countries. While melasma treatment is off-label in Europe, it is widely used based on supporting evidence [25,26,27].

In Romania, AzA is available in both cream (20%) and gel (15%) formulations, officially registered in the Romanian national medicines database. These topical treatments are indicated for acne vulgaris and rosacea and are dispensed as prescription-only medications. The 15% gel formulation is particularly used for papulopustular rosacea, and its presence is confirmed in the national regulatory authority’s Summary of Product Characteristics (SmPC). AzA’s recognized efficacy and tolerability, especially in individuals with sensitive skin, support its use in mild to moderate inflammatory skin conditions [28].

**Figure 2:**
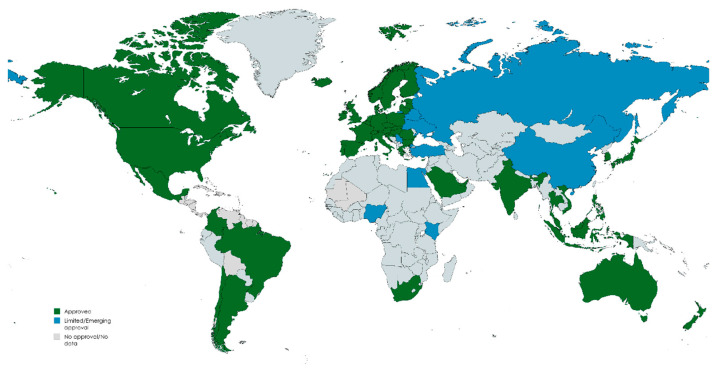
Global market map: usage and regulatory approvals of azelaic acid-based products [29,30,31].

The global market for azelaic acid-based dermatological products has shown steady growth, driven by increasing demand for effective acne, rosacea, and hyperpigmentation therapies. According to Market.us and Allied Market Research, the market was valued at over USD 200 million in 2023 and is projected to exceed USD 415 million by 2033, growing at a CAGR of approximately 7%. This growth is supported by strong market penetration across North America, Europe, and Asia-Pacific, with Europe showing notable demand driven by cosmetic and pharmaceutical sectors. AzA’s dual availability as both a prescription medication and an over-the-counter (OTC) ingredient has further facilitated its widespread clinical adoption. Though specific regulatory approvals vary by region (Figure 2), the global trend supports AzA’s acceptance as a first-line or adjunct treatment. Notably, industry attention is turning to innovative delivery technologies—such as deep eutectic solvent (DES)-based formulations—that aim to enhance solubility, skin penetration, and formulation sustainability, potentially expanding market reach and therapeutic versatility [29,30,31].

Initially, formulations relied on simple creams and gels with propylene glycol or alcohol, but these often-caused irritation or dryness [16,32,33]. Modern delivery systems now include liposomes [19,24], ethosomes [34], solid lipid nanoparticles [35], chitosan hydrogels [36], and cyclodextrin nanosponges [37]—designed to improve absorption and reduce irritation. Among these, DESs have shown strong potential by enhancing solubility, penetration, and formulation sustainability [38,39,40].

In conclusion, AzA is a multifunctional dermatological agent with proven safety, tolerability, and efficacy across acne, rosacea, and pigmentation disorders. Despite solubility challenges, innovations in formulation science—particularly with DESs and nanocarriers—are unlocking new therapeutic opportunities for this established compound.

## 2. Pharmacodynamics and Mechanisms of Action: Biological Activities of Azelaic Acid and Their Therapeutic Relevance

AzA exhibits a diverse array of biological activities that underpin its well-established therapeutic applications in dermatology.

These include antimicrobial, anti-inflammatory, antimelanogenic, and antioxidant mechanisms, each mediated by distinct molecular targets and pathways. An overview of these actions is presented in Table 2 and further explored in the sections that follow.

**Figure 3:**
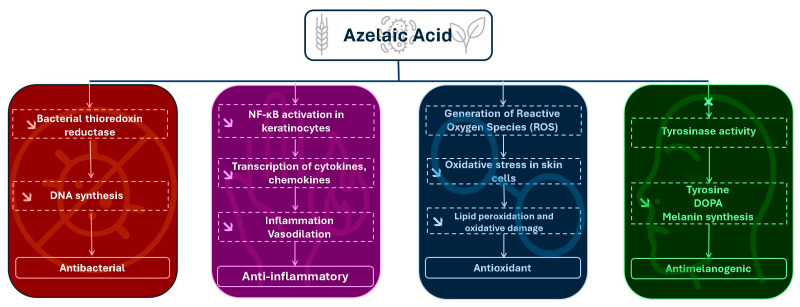
Biological mechanisms of action of azelaic acid.

These diverse mechanisms (Figure 3) of action include antibacterial effects, which help to reduce microbial colonization of the skin [5], anti-inflammatory properties, contributing to the mitigation of inflammatory skin conditions [43], antimelanogenic activity, which plays a role in the modulation of hyperpigmentation disorders [3], and antioxidant effects, offering protection against oxidative stress-induced cellular damage [4]. Each of these bioactivities are mediated by distinct and complex biochemical pathways involving specific molecular targets and signaling cascades. This chapter aims to provide a comprehensive and mechanistic examination of these activities, with a particular focus on the molecular interactions and regulatory networks that govern the therapeutic efficacy of Aza in dermatological contexts.

### Antibacterial Mechanism and Other Medical Indications

The antibacterial activity of AzA is well-documented, particularly against *Cutibacterium acnes* (formerly *Propionibacterium acnes*), the bacterium implicated in acne pathogenesis. The primary mechanism involves the inhibition of bacterial mitochondrial oxidoreductase enzymes, which are essential for microbial respiration and protein synthesis. By targeting this enzymatic system, AzA disrupts energy production within bacterial cells, leading to reduced proliferation and eventual bacterial death [3,4,34,49].

In vitro studies have demonstrated that AzA exhibits antibacterial activity against Gram-positive skin flora, including *Cutibacterium acnes* and *Staphylococcus epidermidis* [34,50]. Minimum inhibitory concentration (MIC) and minimum bactericidal concentration (MBC) evaluations confirmed its efficacy, with ethosomal formulations showing enhanced antibacterial effects compared to conventional creams [34]. Unlike antibiotics, AzA’s mechanism, which involves inhibition of bacterial thioredoxin reductase and DNA synthesis, is associated with a lower risk of inducing bacterial resistance, supporting its use in long-term topical therapies [3].

Additionally, AzA exhibits bacteriostatic activity rather than bactericidal effects at therapeutic concentrations, inhibiting the proliferation of *Cutibacterium acnes* and other Gram-positive skin flora without complete eradication [3]. This selective antimicrobial action is considered less disruptive to the commensal skin microbiome compared to broad-spectrum antibiotics, thereby helping to maintain microbial balance while reducing pathogenic overgrowth [51].

Recent research indicates that AzA may also inhibit biofilm formation by *C. acnes*, reducing bacterial virulence and contributing to its anti-acne efficacy [52,53]. This anti-biofilm activity is thought to involve interference with bacterial adhesion and inhibition of extracellular polymeric substance (EPS) production, compromising the structural integrity of the biofilm matrix [52,54].

Furthermore, the antibacterial efficacy of AzA is influenced by environmental pH, with enhanced activity observed under mildly acidic conditions that reflect the microenvironment of acne-affected skin [3]. This pH-dependence may contribute to its targeted action in diseased skin while sparing healthy regions.

Another mechanistic layer involves AzA’s ability to induce intracellular acidification within bacterial cells by facilitating proton influx, disrupting the proton gradient necessary for bacterial energy metabolism and homeostasis [3].

While AzA shows strong activity against Gram-positive bacteria, its antimicrobial spectrum does not extend effectively to Gram-negative species, highlighting its selective antibacterial profile [3]. AzA exerts its antimicrobial effect by inhibiting microbial intracellular enzymes, rather than targeting the cell wall like many traditional antibiotics—a distinction that may contribute to its lower risk of resistance [55].

AzA’s anti-inflammatory activity is mediated through the downregulation of pro-inflammatory cytokines, including interleukin-1 beta (IL-1β) and tumor necrosis factor-alpha (TNF-α), which are key drivers of inflammation in acne and rosacea pathophysiology. By inhibiting their expression, AzA reduces leukocyte recruitment and inflammatory cascade activation within the dermis [43,56].

In vitro studies using cultured keratinocytes have shown that AzA suppresses nuclear factor kappa B (NF-κB) signaling, a transcription factor that regulates inflammatory gene expression, thereby limiting the transcription of cytokines, chemokines, and adhesion molecules and attenuating local inflammatory responses [43].

Furthermore, AzA exhibits antioxidant effects that help reduce oxidative stress in inflamed skin, a mechanism partly attributed to its suppression of pro-oxidative mediators and inflammatory signaling pathways [43,57]. This antioxidant-linked anti-inflammatory mechanism contributes to decreased erythema and papular formation in inflammatory skin disorders [43,56].

Clinical studies corroborate these mechanistic findings, demonstrating significant reductions in erythema and papule counts in rosacea patients treated with a gel containing 15% AzA, while maintaining a favorable safety profile for long-term use without systemic immunosuppression [56].

Additionally, AzA may modulate the expression of matrix metalloproteinases (MMPs), enzymes involved in extracellular matrix remodeling during inflammation; preliminary studies suggest downregulation of MMP-9 activity, potentially reducing tissue degradation and inflammatory damage [57].

Beyond these actions, AzA has been shown to inhibit the activity of kallikrein 5 and the expression of cathelicidin (LL-37), key contributors to the inflammatory response in rosacea, thereby mitigating inflammatory cascades specific to this condition [43,56]. It also activates peroxisome proliferator-activated receptor gamma (PPARγ) in keratinocytes, promoting an anti-inflammatory gene expression profile and further suppressing pro-inflammatory mediators [43]. Furthermore, AzA may reduce neutrophil chemotaxis, limiting immune cell infiltration into inflamed skin and contributing to its broad anti-inflammatory efficacy [56].

AzA exerts a potent antimelanogenic effect by targeting melanogenesis at the enzymatic level. Its primary mechanism involves competitive inhibition of tyrosinase, a copper-containing enzyme responsible for catalyzing the conversion of tyrosine to dihydroxyphenylalanine (DOPA) and ultimately to melanin [58,59,60]. By binding to the active site of tyrosinase, reduces melanin synthesis, leading to skin lightening effects in hyperpigmented lesions [58,59,60].

In vitro enzyme kinetics studies have demonstrated a dose-dependent inhibition of tyrosinase activity by AzA, with reported IC_50_ values ranging approximately from 1 to 2 mM depending on assay condition [59,60,61,62]. This competitive inhibition is reversible, distinguishing it from irreversible tyrosinase inhibitors such as hydroquinone [58,59].

Additionally, AzA selectively inhibits hyperactive melanocyte proliferation and DNA synthesis without significantly affecting normal melanocytes [58,63]. This selective cytotoxicity contributes to its safety in treating melasma and post-inflammatory hyperpigmentation, reducing the risk of hypopigmentation in adjacent healthy skin [58,63].

Histological analyses of hyperpigmented skin treated with AzA demonstrate a reduction in melanocyte activity and melanin granule deposition in the basal epidermis [58,63]. Clinical trials corroborate these findings, showing significant reductions in melanin index and visible pigmentation after 12–16 weeks of topical AzA therapy [58,63].

Notably, AzA’s antimelanogenic effect is effective as monotherapy and does not require corticosteroid or tretinoin co-administration [58,63].

AzA is increasingly acknowledged for its antioxidant role alongside its anti-inflammatory and antimicrobial effects [4,42,64]. It acts by reducing the generation and activity of reactive oxygen species (ROS), which are key contributors to oxidative stress in inflammatory and photodamaged skin [42,64].

Evidence from in vitro and clinical research demonstrates that AzA lowers oxidative stress markers and protects skin cells from lipid peroxidation and oxidative injury, thereby supporting skin barrier integrity [4,64].

Moreover, its ROS-scavenging capacity is believed to complement endogenous antioxidant systems, such as glutathione and superoxide dismutase, although direct upregulation by AzA requires further confirmation [4,42].

The antioxidant properties of AzA contribute to its therapeutic efficacy in inflammatory skin conditions like acne and rosacea, by reducing oxidative tissue damage and preventing melanocyte stimulation that leads to post-inflammatory hyperpigmentation [42,64]. While AzA may be compatible with other antioxidant agents like vitamin C or niacinamide in combination treatments, synergistic effects have yet to be fully substantiated [4].

The main pharmacological targets and mechanisms of AzA in dermatological applications are summarized in Table 3.

## 3. Clinical Studies

### 3.1. Clinical Trials and Outcomes in Acne Vulgaris

AzA has been extensively evaluated in randomized controlled trials for the treatment of acne vulgaris due to its antibacterial, anti-inflammatory, and anti-keratinizing properties. Thielitz et al. [17] conducted a parallel-group study comparing AzA 15% gel to adapalene 0.1% gel in female adult acne patients over a 9-month period, finding comparable efficacy in lesion reduction with significantly lower irritation scores in the AzA group.

Similarly, Katsambas et al. [67] performed a double-blind clinical trial comparing 20% AzA cream with 0.05% tretinoin cream and a placebo vehicle, reporting that AzA reduced both inflammatory and non-inflammatory lesion counts with fewer local side effects compared to tretinoin.

In another randomized clinical trial, Iraji et al. [68] evaluated 20% AzA gel in 60 patients with mild-to-moderate acne vulgaris, demonstrating significant reductions in total lesion counts and improvement in acne severity scores after 12 weeks of treatment, with minimal adverse effects. Collectively, these studies support AzA as an effective and well-tolerated topical therapy for mild-to-moderate acne vulgaris.

### 3.2. Rosacea: Evidence-Based Efficacy

AzA has been extensively studied as a topical treatment for inflammatory skin conditions such as papulopustular rosacea, owing to its antibacterial and anti-inflammatory properties targeting *Cutibacterium acnes* proliferation and keratinocyte hyperproliferation. A randomized, double-blind trial by Maddin compared AzA 20% cream to metronidazole 0.75% cream in patients with papulopustular rosacea over 15 weeks, demonstrating similar reductions in inflammatory lesions, with AzA achieving greater erythema improvement and fewer stinging and burning sensations [69].

A pivotal randomized, double-blind trial by Thiboutot et al. [70] showed that 15% AzA gel applied twice daily for 12 weeks resulted in a 67.2% reduction in inflammatory lesions compared to a 46% reduction with placebo (*p* < 0.001), alongside significant improvements in erythema scores.

Further evidence from Elewski et al. [15] confirmed the superior performance of 15% AzA gel compared to 0.75% metronidazole gel, showing a 72.7% lesion reduction versus 55.8% (*p* < 0.001) and better erythema outcomes over 15 weeks in 251 patients. Additionally, 69% of patients treated with AzA were rated as “clear” or “almost clear” on the Investigator Global Assessment scale, compared to 55% of those treated with metronidazole (*p* = 0.02). Erythema improved in 56% versus 42% of patients, respectively, (*p* = 0.02).

A comprehensive review by Frampton and Wagstaff summarized the results of three pivotal randomized, double-blind, multicenter clinical trials evaluating AzA 15% gel for papulopustular rosacea. In two vehicle-controlled trials (*n* = 329 and *n* = 335), AzA demonstrated a significantly greater reduction in inflammatory lesion counts compared to vehicle, with mean decreases of 58% and 51% at 12 weeks (*p* = 0.0001 and *p* = 0.0208, respectively). Improvement in erythema severity was also superior with AzA showing 44–46% improvement vs. 28–29% with vehicle (*p* = 0.0017 and *p* = 0.0005). Furthermore, a head-to-head trial involving 251 patients comparing AzA with 0.75% metronidazole gel demonstrated that AzA produced greater reductions in inflammatory lesions (−73% vs. −56%; *p* < 0.001) and erythema improvement (56% vs. 42%; *p* = 0.02) after 15 weeks, with both treatments demonstrating comparable tolerability and cosmetic acceptability [71].

Clinical trials consistently demonstrate that AzA 15% gel provides significant reductions in inflammatory lesions and erythema in papulopustular rosacea, with efficacy comparable to or exceeding that of metronidazole 0.75% gel, and with favorable tolerability and cosmetic outcomes reported across multiple randomized controlled trials.

### 3.3. Hyperpigmentation Disorders: Monotherapy and Combination Regimens Clinical Trials in Hyperpigmentation Disorders

A randomized, double-masked, multicenter trial evaluated AzA 20% cream versus vehicle in patients with Fitzpatrick skin phototypes IV to VI presenting facial hyperpigmentation. Over a 24-week period, AzA produced significantly greater reductions in pigmentary intensity than vehicle, as measured by both an investigator-rated scale (*p* = 0.021) and chromometer analysis (*p* = 0.039). Global improvement scores also significantly favored AzA (*p* = 0.008). Although transient burning (*p* ≤ 0.046) and stinging (*p* = 0.002) were more frequent during early treatment, the overall tolerability was good, with no discontinuations due to adverse effects. Patients treated with AzA more frequently report smoother skin, higher satisfaction, and better perceived efficacy compared to previous treatments. These results underscore AzA’s efficacy and favorable cosmetic acceptability for managing hyperpigmentation in individuals with skin of color [72].

Extensive clinical research has established AzA as a safe and effective agent for treating various pigmentary disorders, particularly melasma and postinflammatory hyperpigmentation. Its therapeutic utility in dermatology is supported by several well-structured clinical trials assessing its role both as monotherapy and in combination regimens.

More directly focused on hyperpigmentation, a randomized, double-masked, multicenter study evaluated AzA 20% cream in darker-skinned individuals (Fitzpatrick phototypes IV to VI) with facial hyperpigmentation. Over a 24-week period, AzA significantly reduced pigmentary intensity compared to vehicle, as measured by both the investigator’s subjective scale (*p* = 0.021) and objective chromometer analysis (*p* = 0.039). Global improvement scores were also significantly higher with AzA (*p* = 0.008). Patients reported smoother skin, greater satisfaction, and improved perceptions of efficacy compared to past treatments. Mild to moderate burning (weeks 4 and 12) and stinging (week 4) were reported more frequently with AzA, but these symptoms were transient and the treatment was generally well tolerated [72].

A comparative open-label trial in Pakistan assessed the efficacy of AzA 20% cream alone versus its combination with tretinoin 0.1% cream in 48 patients with melasma. After six months of treatment, 79% of patients in the combination group showed excellent or good improvement, compared to 71% in the monotherapy group; both results were statistically significant (*p* < 0.05), although no direct comparison between groups was reported. Notably, the combination therapy led to earlier onset of visible lightening-by the sixth week versus the ninth week in the monotherapy group. Tolerability was high in both arms, with only transient mild irritation reported in about 10% of patients [73].

Collectively, these clinical trials underscore AzA’s robust efficacy and favorable safety profile in the treatment of hyperpigmentation disorders. Its versatility, whether used alone or in synergistic combinations with agents like glycolic acid or tretinoin, makes it particularly suitable for diverse patient populations, including those with darker skin tones who are often more prone to postinflammatory hyperpigmentation. As a non-hydroquinone alternative, AzA remains a cornerstone in pigmentary disorder management.

### 3.4. Safety and Tolerability Profile

AzA is generally well tolerated; though, clinical trials and dermal safety studies have consistently reported mild to moderate local adverse effects. The most common include burning, stinging, pruritus, erythema, dryness, and scaling, particularly during the initial weeks of therapy [1,71]. In a randomized controlled trial comparing AzA 20% cream with hydroquinone 4% for melasma, Gupta et al. [74] observed burning and stinging in 1% to 5% of patients. These effects were mild, transient, and did not lead to discontinuation.

Similarity, a 24-week study by Kakita et al. [75] involving AzA 20% (combined with 15–20% glycolic acid) reported mild, transient local irritation, slightly more frequent than with hydroquinone 4%; yet, all symptoms remained within the “trace” to “mild” range. No participants discontinued treatment, and completion rates were high, highlighting its good tolerability even in darker-skinned patients.

In rosacea, comparative studies between AzA 15% gel and metronidazole 0.75% gel consistently demonstrated a favorable safety profile. While neurosensory symptoms such as stinging, tingling, and burning were reported slightly more often with AzA, they were usually transient and resolved within one to two weeks [18,76].

Rare adverse effects, such as contact dermatitis, facial edema, and acne occur in fewer than 1% of patients and are typically mild and reversible upon discontinuation of treatment [1,71].

Importantly, systemic side effects are exceedingly rare due to minimal percutaneous absorption [1]. No systemic toxicity has been reported with topical AzA use, and it is considered safe during pregnancy, supporting its favorable safety profile in reproductive-age women [77].

A randomized trial by Tehrani et al. [78] comparing AzA 20% plus hydroquinone 5% versus hydroquinone alone in melasma found burning and stinging to be more frequent in the combination group (50% vs. 35%; *p* = 0.034), though symptoms were mostly mild and well tolerated. Similarly, King et al. [19], in a systematic review, reported a slightly higher incidence of burning with AzA than hydroquinone, but both treatments were considered safe and effective.

Table 4 allows the evaluation of the efficiency of different formulations containing AzA compared to other dermatological treatments, based on the reported literature data.

## 4. Stability and Analytical Methods

### 4.1. Introduction and Analytical Challenges

AzA presents notable analytical challenges due to its non-chromophore structure lacking strong UV-absorbing or fluorescent groups. As a result, accurate quantification in pharmaceutical and cosmetic formulations often requires chemical derivatization to introduce detectable groups or improve volatility. However, most of these methods require chemical derivatization of AzA to introduce a chromophore or improve volatility-for example, pre-column tagging with fluorescent reagents (e.g., leucine–coumarinylamide or 4-bromophenacyl bromide) or silylation/methylation for GC analysis. While derivatization enhances sensitivity, it introduces significant limitations: time-consuming procedures, demand of advanced chemistry expertise, unsatisfactory chemical stability of derivatives (mono- or di-esters) that lead to peak broadening and poor reproducibility. These challenges have spurred the development of non-derivatized and stability-indicating analytical methods, which are now increasingly favored for AzA quantification in quality control and formulation development contexts [85].

### 4.2. Stability Testing of Azelaic Acid Formulations

Stability studies assess whether a formulation maintains its chemical potency and physical integrity under defined environmental conditions. ICH guidelines (e.g., Q1A/R2) recommend testing at controlled temperatures/humidities (e.g., long-term 25 °C, accelerated 40 °C) to simulate shelf-life. Acceptable stability is typically defined as retention of 90–110% of the initial drug content, without significant degradation or physical change. For example, an ethosome-based AzA cream stored over 12 weeks exhibits minimal degradation, maintaining drug content within regulatory limits alongside stable pH, viscosity, and physical appearance. Conversely, stress testing-exposing AzA to heat, light, oxidation, or pH extremes-often reveals degradation pathways. Notably, during accelerated testing of a liposomal AzA formulation, a previously unreported impurity was identified as ethyl azelate, likely formed through esterification between AzA and the ethanol excipient [86]. This finding underscores the critical need for stability-indicating methods capable of differentiating AzA from its degradation products and excipient-derived by-products.

### 4.3. Stability-Indicating Analytical Methods

According to ICH Q2(R1), a stability-indicating method must specifically quantify the intact active pharmaceutical ingredient (API) in the presence of impurities, degradants, or excipients. Method development typically involves forced degradation of AzA, followed by validation of the method’s capacity to resolve and quantify the parent compound.

High-performance liquid chromatography (HPLC) is the method of choice. In one validated study, Malik et al. [85] demonstrated a reversed-phase HPLC-UV method using a C18 column and a phosphate buffer–acetonitrile mobile phase (75:25, pH 3.5), detecting AzA at 206 nm. The method showed excellent specificity, with no signal from placebo matrices and clear resolution of degradation peaks. It remained robust across stress conditions (freeze–thaw, solution aging) and consistently recovered >96% of AzA, confirming suitability for stability and routine testing.

### 4.4. Chromatographic Techniques for Azelaic Acid Analysis

Reversed-phase HPLC remains the most widely used and validated approach for AzA. Historically, pre- or post-column derivatization was employed to enhance UV detection. However, recent methods capitalize on AzA’s weak absorbance at ~204–210 nm, using acidic mobile phases to enhance signal and enable direct detection. For example, a validated isocratic HPLC method using UV detection at 206 nm achieved a retention time of ~8 min and was linear over 5–400 μg/mL (r^2^ = 0.998), with good recovery from commercial 10% AzA creams (>97%) and RSD values < 2%, fulfilling ICH validation criteria [85].

Alternative detectors such as evaporative light scattering (ELSD) and charged aerosol detection (CAD) have also been employed to circumvent AzA’s weak UV absorbance. ELSD enabled impurity detection in liposomal formulations, while LC-MS/MS methods—operated in negative ion mode—offer superior selectivity and sensitivity (ng/mL range), particularly valuable for biological matrices or complex extracts [7,86]. However, cost and instrumentation complexity limit routine LC-MS use in standard quality control labs. Gas chromatography (GC), though less common, is used for AzA trace analysis in biological and forensic applications. Due to AzA’s non-volatility, derivatization (e.g., to TMS or methyl esters) is essential. GC-MS has proven effective for identifying AzA and its degradants—such as ethyl azelate—especially in stability testing or impurity profiling [85,86].

### 4.5. Spectrophotometric Assays for Azelaic Acid

Despite AzA’s weak UV activity, direct UV spectrophotometry remains viable at low wavelengths (e.g., 204 nm), particularly for bulk materials or high-concentration dosage forms. One validated method using phosphate buffer (pH 6.8) demonstrated linearity in the 10–50 μg/mL range and recovery rates between 98 and 102%, with minimal excipient interference [87]. However, this technique offers lower sensitivity and higher susceptibility to baseline noise compared to HPLC and requires rigorous blank correction.

Although colorimetric assays exploiting AzA’s reactivity remain unexplored, they represent a promising avenue for simplified field or low-resource settings in future research.

### 4.6. Method Validation and Performance Parameters

All analytical methods for AzA must comply with ICH Q2(R1) validation guidelines, addressing:

- Specificity: ability to differentiate AzA from excipients and degradants.
- Linearity: typically validated over 2–3 orders of magnitude (e.g., 5–400 μg/mL).
- Accuracy and Precision: recovery of 97–100% and %RSD ≤ 2% in replicate tests.
- LOD/LOQ: for the UV-HPLC method at 206 nm, LOD was ~1.08 μg/mL and LOQ 3.28 μg/mL [85]; LC-MS techniques may offer sub-ng/mL limits.
- Robustness: demonstrated stability under ±10% variations in pH, flow rate, and detection wavelength.

## 5. Formulation Strategies and Delivery Systems: From Conventional Vehicles to Advanced Nanocarriers

Topical delivery of AzA is limited by its poor aqueous solubility (~2.4 mg/mL at 25 °C) [88] high melting point (106.5 °C), and moderate lipophilicity (log P ~1.57) [2], which collectively hinder passive diffusion through the stratum corneum [88]. Formulation strategies have thus focused on enhancing solubilization, improving skin penetration, increasing cutaneous retention, and minimizing local irritation.

### 5.1. Conventional Formulations (Gels, Creams, Foams)

AzA has been formulated into various conventional dosage forms, including gels [70,89], creams [90], and foams [91,92], to optimize topical delivery, enhance patient compliance, and balance therapeutic efficacy with tolerability. These formulations differ in their rheological properties, excipient composition, and skin permeation profiles [93].

The earliest commercial formulation was a 20% AzA cream, approved in Europe for acne vulgaris and rosacea [94].

This cream employs a hydrophilic base containing propylene glycol [94], as a penetration enhancer, which facilitates stratum corneum permeation. It also includes benzoic acid as a preservative [95], as well as cetostearyl alcohol for consistency and a long-term stability [96], and purified water, resulting in a semisolid emulsion with a pH of 4.8 [97].

Recent studies have explored semi-solid emulsions and oil-in-water creams with modified emulsifier systems to further optimize skin penetration while minimizing irritation [50]. Formulations containing polyethylene glycol stearate [98] and cetearyl alcohol have demonstrated improved occlusivity and enhanced skin hydration compared to traditional creams, potentially reducing barrier disruption commonly associated with AzA.

Overall, conventional formulations of AzA continue to evolve through modulation of excipients and delivery systems to improve patient adherence, reduce irritation, and enhance therapeutic outcomes.

### 5.2. Nanocarrier-Based Formulations

Beyond conventional creams and gels, innovative nanocarrier systems have been developed to overcome AzA’s low aqueous solubility, limited skin penetration, and potential for local irritation. These advanced delivery systems enhance drug localization in the epidermis, promote sustained release, and minimal systemic exposure [99]. Key nanocarriers investigated include liposomes [100], ethosomes [101], niosomes [102], nanostructured lipid carriers (NLC) [103], nanosponges [37]. Emerging platforms, such as polymeric nanoparticles and solid lipid nanoparticles, are also being explored to further improve drug retention, skin compatibility, and therapeutic efficacy.

#### 5.2.1. Liposomes

**Figure 4:**
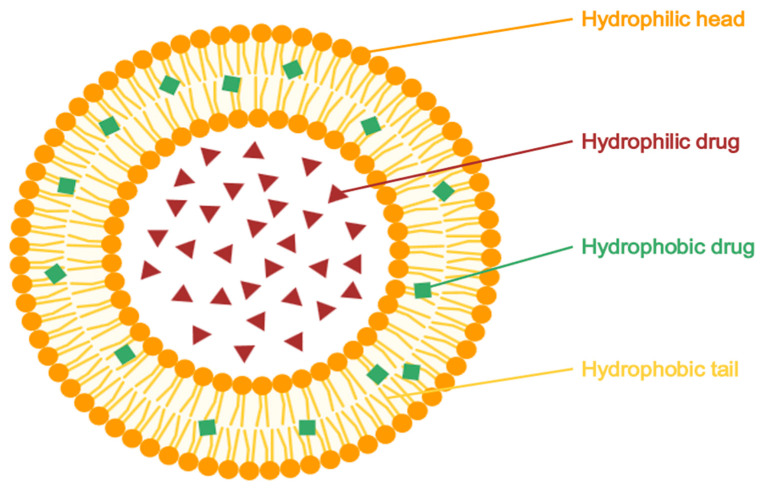
Liposomes as spherical vesicles with phospholipid bilayers, enabling targeted delivery of hydrophilic and lipophilic drugs to skin layers while reducing systemic exposure.

Liposomes (Figure 4) are small, spherical vesicles composed of one or more phospholipid bilayers, capable of encapsulating both hydrophilic and lipophilic drugs. They present versatile carriers in topical drug delivery systems due to their biocompatibility, ability to control drug release, and potential to enhance skin targeting. Liposomes can localize active pharmaceutical ingredients (APIs) within the specific skin layers, such as the stratum corneum or appendages, thereby minimizing systemic exposure. Their performance is strongly influenced by vesicle size and lipid membrane composition, which affect both skin interaction and drug permeation [24,104].

A 2016 study by Burchacka et al. [24] developed two liposomal gel formulation (F1 and F2) containing 10% AzA using soybean phospholipids (Phospholipon 50 IP), carbopol, and other standard excipients. Despite the lower AzA concentration compared to the commercial 20% cream, the F2 formulation achieved markedly greater drug accumulation in the stratum corneum (187.5 mg/cm^2^ vs. 52.3 mg/cm^2^). The F2 formulation, optimized by a two-step addition of AzA, produced liposomes with a mean diameter of ~211 nm and a polydispersity index (PDI) below 0.25, indicating a homogeneous size distribution. Stability studies under ICH guidelines confirmed its physical and microbiological stability over six months. Notably, the intrinsic antimicrobial properties of AzA eliminated the need for preservatives. These findings support the potential of liposomal formulations to improve cutaneous bioavailability, reduce irritation, and enhance therapeutic efficacy in conditions such as acne, rosacea, and hyperpigmentation [24].

In another study, Pasca et al. [100] prepared liposomes composed of phosphatidylcholine and cholesterol, incorcorating15–25% AzA by a lipid-film hydration method. The 20% formulation (AALipo 20%) showed optimal performance, with vesicle diameters below 500 nm, a zeta potential of approximately −18 mV, and an encapsulation efficiency of 85.7%, indicating colloidal stability and high drug loading. Compared to free AzA in cream or solution, the liposomal formulation exhibited superior antimicrobial activity against *Staphylococcus aureus* (26 mm vs. 23 mm inhibition zone) and *Enterococcus faecalis*, maintained dermal fibroblast viability, promoted complete scratch-wound closure within 36 h, and mitigated oxidative DNA damage in comet assays. These results underscore the improved bioactivity, biocompatibility, and skin-targeting potential of liposomal AzA for topical acne management [100].

#### 5.2.2. Ethosomes

**Figure 5:**
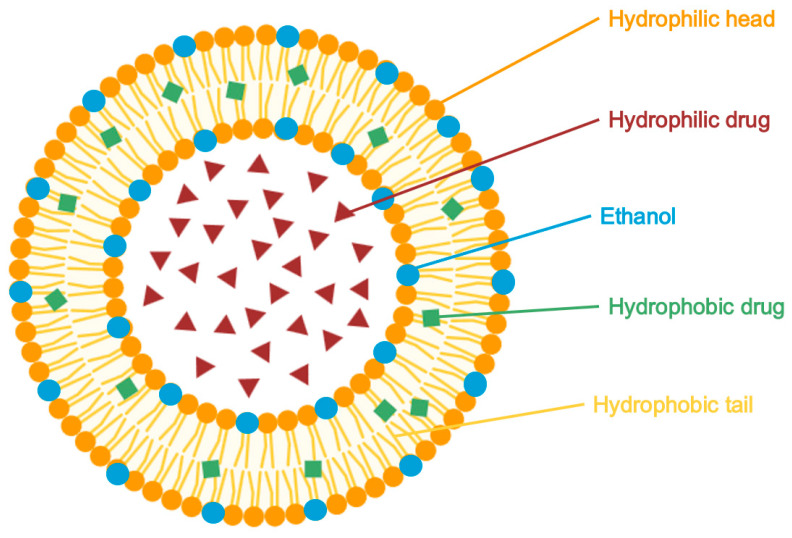
Ethosomes as flexible, ethanol-rich vesicles that enhance skin permeation, improve drug localization, and offer sustained release with reduced systemic absorption.

Ethosomes (Figure 5) are flexible, phospholipid-based vesicles containing 20–45% ethanol, which acts as a potent permeation enhancer. Ethanol disrupts the intercellular lipid architecture of the stratum corneum, increasing membrane fluidity and allowing the vesicles to deform and penetrate deeply through the skin layers. This alcohol-induced intercalation also imparts a net negative surface charge to the vesicles, reducing aggregation and improving colloidal stability compared to conventional liposomes. When applied topically, ethosomes improve drug localization within the stratum corneum and viable epidermis, reduce systemic absorption, and enable sustained drug release. These properties lead to enhanced bioavailability prolonged skin retention, reduced toxicity, and improved clinical efficacy, making ethosomes a promising system for the topical delivery of anti-acne agents [105].

In a 2016 study, Mistry and Ravikumar developed an azelaic acid-loaded ethosomal formulation optimized for acne treatment. Using thin-film hydration (outperforming hot and cold methods), they formulated vesicles with soya phosphatidylcholine, cholesterol, and ethanol, with component ratios optimized via central composite design. The resulting ethosomal vesicles exhibited a mean size of 514.3 nm, a low polydispersity index (PDI = 0.08), and a zeta potential of −35.44 mV, indicating a stable and uniform dispersion. Entrapment efficiency reached 91.86 ± 2.25%, and the ethosomal gel exhibited a skin-compatible pH (5.6) and high drug content (98.2%). In vitro and ex vivo studies revealed sustained drug release over 12 h (89.6% and 93.4%, respectively), surpassing both a conventional gel and a marketed cream. The formulation followed zero-order release kinetics (R^2^ = 0.9819), achieved a flux of 25.54 mg/cm^2^/h, and demonstrated superior antimicrobial activity (2.5 cm inhibition zone). Rheological assessments confirmed favorable spreadability and viscosity, and stability was maintained over three months at room temperature. These findings support the ethosomal gel as a non-irritant, effective, and stable carrier for AzA in topical acne therapy [105].

In another study, Apriani et al. [34] prepared AzA ethosomes using Phospholipon 90 G (a natural phospholipid) and cholesterol, casting a lipid film from dichloromethane-methanol (2:1), followed by hydration with an ethanol/phosphate-buffer solution (30–40% ethanol) containing AzA. The resulting ethosomal suspension was incorporated into a 20% cream base composed of stearic acid, cetyl alcohol, isopropyl myristate, glyceryl monostearate, glycerine, propylene glycol, and triethanolamine. Among three ethanol concentrations tested, the 35% batch (ETHO 35) was optimal, yielding vesicles with a diameter of 179 ± 2 nm, PDI of 0.665, zeta potential of −34.9 mV, and encapsulation efficiency of 94.5%. The final cream was stable, white, homogeneous, slightly acidic (pH 4.90), and exhibited appropriate viscosity (~4900 cP), with a measured AzA content of 21% *w*/*w*. In microbiological testing against Propionibacterium acnes, both the ethosome cream and a commercial non-vesicular product exhibited a minimum inhibitory concentration (MIC) of 250 µg/mL. However, ETHO 35 demonstrated a lower minimum bactericidal concentration (MBC: 250 vs. 500 µg/mL) and reduced colony formation at sub-MIC, indicating superior antimicrobial efficacy [34].

#### 5.2.3. Niosomes

**Figure 6:**
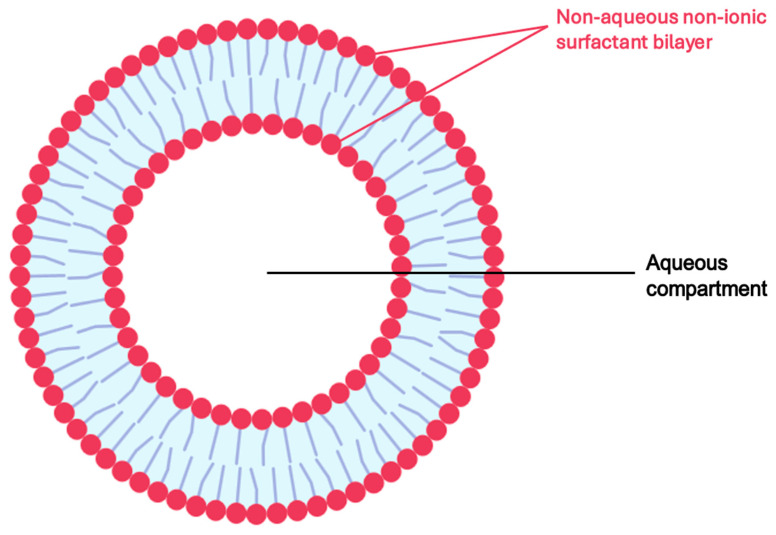
Niosomes as non-ionic surfactant-based vesicles offer enhanced skin penetration, prolonged drug release, and improved topical bioavailability.

Niosomes (Figure 6) are microscopic, non-ionic surfactant-based vesicles composed of a bilayer structure that can encapsulate both hydrophilic and lipophilic drugs, functioning as stable and flexible drug delivery systems. Unlike liposomes, which are composed of phospholipids, niosomes are formed using non-ionic surfactants, often in combination with cholesterol, for enhanced membrane stability. Their advantages in topical and transdermal drug delivery include improved drug skin penetration, through the stratum corneum, prolonged release profiles, reduced systemic absorption, and increased drug bioavailability at the target site [106,107,108].

In a 2023 study, Kashyap and Rani developed a niosomal gel formulation of AzA for acne therapy using the thin-film hydration technique. Niosomes were prepared using various non-ionic surfactants (Span 20, 40, and 60) and cholesterol, with aloe vera incorporated to mitigate skin irritation. The optimized niosomal formulations were incorporated into a Carbopol 940 gel base containing 2% *w*/*w* AzA. Among 15 tested formulations, F9 (Span 40-based) demonstrated the highest entrapment efficiency (72.3%) and drug release (82.69% over 24 h), outperforming formulation with Span 20 and 60. Particle sizes ranged from 226 to 272 nm, and scanning electron microscopy confirming spherical morphology. In vitro diffusion studies demonstrated sustained drug release, with F15 gel achieving 75.59% release at 24 h compared to 60.87% for plain AzA gel. Drug release kinetics followed first-order and Korsmeyer–Peppas models, indicating anomalous non-Fickian diffusion, likely due to combined diffusion and matrix erosion mechanisms. Stability testing under refrigerated conditions (4–8 °C) over 45 days confirmed consistent drug content and preserved physical characteristics. These results highlight Span 40-based niosomal gels as a promising carriers for the controlled, skin-targeted delivery of AzA, offering enhanced therapeutic efficacy and reduced irritation potential for topical acne treatment [102].

#### 5.2.4. Solid Lipid Nanoparticles (SLN)

**Figure 7:**
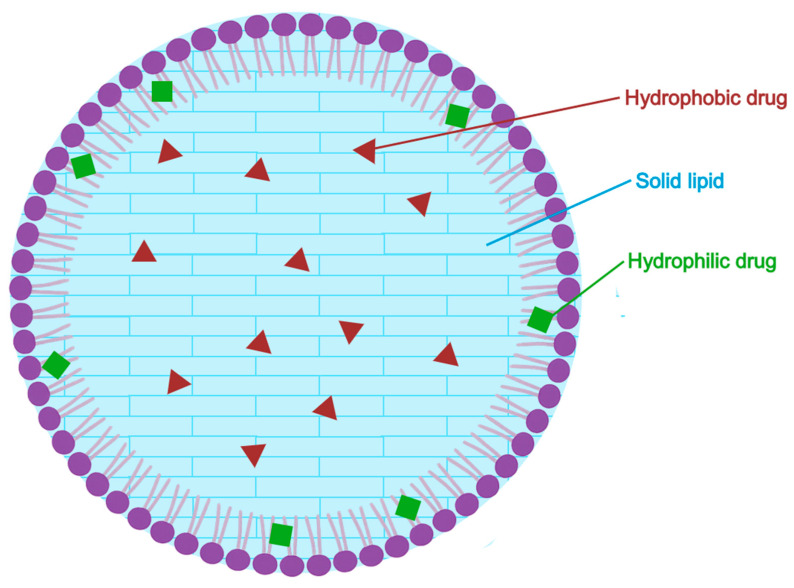
Solid lipid nanoparticles (SLNs) as submicron carriers with a solid lipid core and surfactant shell, enabling stable, biocompatible, and scalable drug delivery with controlled release.

Solid lipid nanoparticles (SLNs), presented in Figure 7, are submicron spherical carriers (typically 40–1000 nm in diameter) composed of lipids that remain solid at both room and physiological temperature. Each nanoparticle has a solid lipid core that solubilizes both hydrophilic and lipophilic drugs, surrounded by a surfactant layer that stabilizes the aqueous dispersion. Developed in the mid–1990s as physically stable and biocompatible alternatives to emulsions, liposomes and polymeric nanoparticles. Their solid core protects the encapsulated drug and can be engineered for sustained or controlled release. Commonly used GRAS (Generally Recognized as Safe) lipids such as stearic acid, glyceryl behenate or tristearin while surfactants like Poloxamer 188 and Tween 80 and ensure colloidal stability. SLNs are typically produced using high-pressure homogenization or high-shear mixing techniques, without the need for organic solvents-facilitating scalability and industrial feasibility [109,110].

In a recent study, Nautiyal and Wairkar formulated azelaic-acid loaded SLNs using ethanol evaporation followed by high-pressure homogenization. The optimized formulation (M4) contained 20% stearic acid, 10% AzA, and Poloxamer 188 as the surfactant-selected based on solubility screening as the most compatible combination. Dynamic light scattering (DLS) and transmission electron microscopy (TEM) analysis revealed nearly spherical particles with a mean diameter of 281–282 nm, polydispersity index (PDI) of ~0.32, and zeta potential of −15 mV. Drug content was 97%, with an encapsulation efficiency of 76.6%.

Franz-diffusion cell experiments demonstrated a 24–h cumulative drug release of 93.9% from the SLNs significantly outperforming the 67.0% release from a marketed 20% cream. Antioxidant capacity measured by DPPH assays showed 70.1% radical scavenging—comparable to the commercial product (86.9%). In B16F10 melanocyte assays, 10% SLNs inhibited melanin production (55.8%) and tyrosinase activity (28.3%) as effectively as the 20% cream, while maintaining higher cell viability (99% vs. 93.7%). Importantly, HET-CAM assays revealed no vascular irritation with the SLNs, unlike the reference cream, which caused slight hemorrhage and coagulation. Stability testing showed minimal particle size increase after 3 months at 5 °C (to 297 nm, ζ ≈ −15 mV), indicating good physical stability. However, storage at room temperature led to greater size growth (up to 392 nm). Overall, the results suggest that 10% azelaic-acid SLNs can achieve depigmenting efficacy comparable to a 20% commercial cream, while reducing cytotoxicity and irritation-offering a dose-sparing, safer alternative for the topical treatment of hyperpigmentation [35].

#### 5.2.5. Nanostructured Lipid Carriers (NLC)

**Figure 8:**
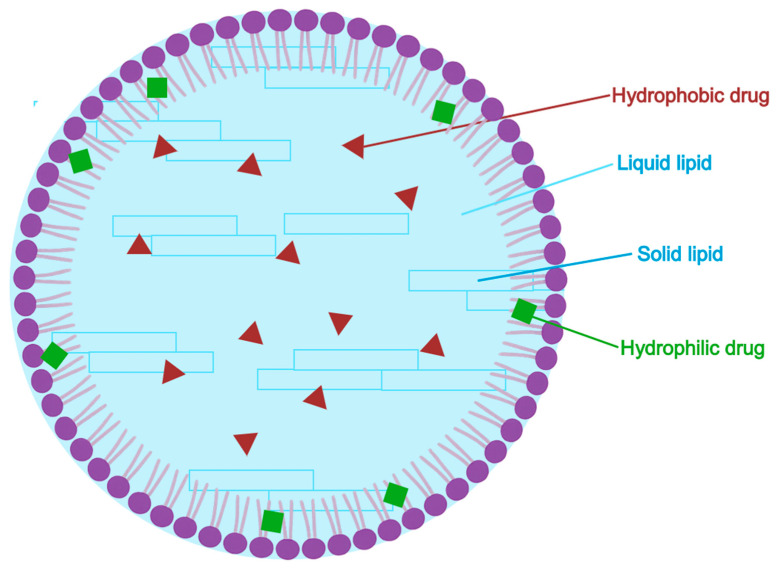
Nanostructured lipid carriers (NLCs) composed of a blend of solid and liquid lipids for improved drug loading, stability, and controlled release in topical delivery.

Nanostructured lipid carriers or NLCs (Figure 8) are advanced lipid-based nanoparticles engineered by blending solid and liquid lipids in a surfactant-stabilized matrix. Developed as a second-generation improvement over SLNs (solid lipid nanoparticles), NLCs address key limitations of SLNs—such as limited drug loading and risk of drug expulsion during storage—by introducing structural imperfections that increase payload accommodation and physical stability. Their partially disordered lipid matrix permits higher drug incorporation, controlled release, and superior skin penetration, making them especially attractive for topical applications [111,112,113].

In a comprehensive 2015 review, Naseri et al. [110] examined the physicochemical characteristics, fabrication techniques, and therapeutic potential of NLCs, particularly in dermal drug delivery. The authors described three principal structural models: (i) imperfect crystal type, characterized by matrix defects allowing drug incorporation; (ii) amorphous-type: a non-crystalline matrix that inhibits drug expulsion and crystallization; (iii) multiple-type: where drugs are solubilized predominantly in the liquid lipid phase, enabling reservoir-like release.

NLCs are typically produced via high-pressure homogenization (hot and cold), ultrasonication, solvent evaporation, and spray drying, with each offering specific advantages regarding particle size control, scalability, and thermal sensitivity. Compared to solid lipid nanoparticles (SLNs), NLCs exhibit greater encapsulation efficiency, enhanced colloidal stability, and more prolonged and consistent drug release profiles. Crucially, NLCs demonstrate high biocompatibility and skin affinity, mimicking the composition of stratum corneum lipids and enhancing cutaneous permeation without compromising barrier function. This makes them ideal carriers for lipophilic active pharmaceutical ingredients (APIs) such as AzA, which typically suffer from poor aqueous solubility and limited skin retention. The review highlights the promise of NLC-based formulations in the treatment of acne and other dermatological conditions, noting their ability to enhance therapeutic efficacy while minimizing irritation and systemic exposure [110].

#### 5.2.6. Nanosponges

Nanosponges are nano-sized (approximately 250 nm to 1 μm), porous, three-dimensional particulate systems formed by cross-linking polymers—most often cyclodextrins—into a “sponge-like” matrix with internal cavities capable of encapsulating both hydrophobic and lipophilic molecules. These nanostructures offer several advantages: they improve drug solubility, provide protection from degradation, enable controlled release, and can modulate skin permeability, making them highly suitable for topical delivery of poorly water-soluble drugs like AzA [114,115,116,117].

Kumar and Rao synthesized β-cyclodextrin nanosponges by melt-blending β cyclodextrin with diphenyl carbonate at 100 °C for 5 h, followed by freeze-drying AzA into the porous network. The 1:6 CD ratio yielded an optimal formulation (AANS 1:6), which enhanced aqueous solubility five-fold (9.28 µg/mL vs. 1.97 µg/mL for free AzA. The resulting particles were spherical, highly porous, and had a mean diameter of 228 nm, a low polydispersity index (PDI = 0.14), and a zeta potential of −21 mV. The encapsulation efficiency reached 88%, indicating strong drug-loading capability.

In vitro release studies in pH 5.5 buffer showed controlled release without burst effect, with roughly approximately 66% of AzA released over 24 h. Cell viability assays in HaCaT keratinocytes showed a higher growth-inhibition rate for the nanosponge (54.9%) at 320 µg/mL compared to free drug (37.2%), but the slightly elevated IC_50_ suggested no increase in overall cytotoxic risk.

Antibacterial testing showed that the nanosponge formulation exhibited double the antimicrobial potency: MIC/MBC values against *Staphylococcus aureus* and *Cutibacterium acnes* were 50/100 µg/mL, compared to 100/200 µg/mL for free AzA—comparable to dapsone controls. Although the nanosponge showed slightly reduced antioxidant activity (64% vs. 71%) and attenuated tyrosinase inhibition (IC_50_ = 4.76 mM vs. 0.81 mM for free drug), the overall profile supports its efficacy as a low-irritant, high-bioavailability carrier system. The study concludes that β-cyclodextrin nanosponges significantly improve solubility, prolong drug release, enhance antimicrobial performance, and reduce irritancy, thus representing a promising and scalable nanocarrier for AzA in the topical management of acne and hyperpigmentation [37].

#### 5.2.7. Nanoemulsions

**Figure 9:**
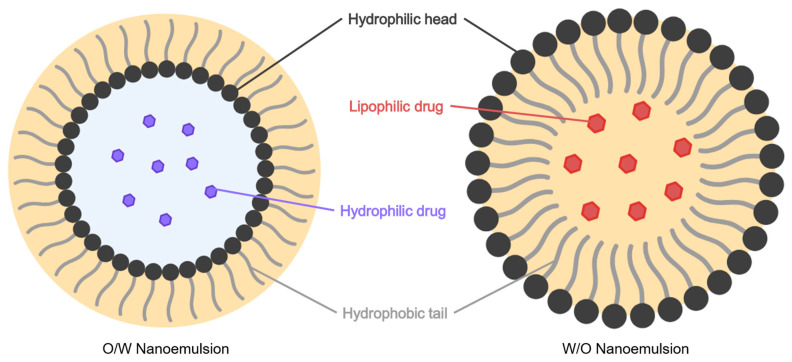
Graphical representation of nanoemulsions.

Nanoemulsions (Figure 9) are kinetically stable, submicron-sized colloidal dispersions composed of oil, water surfactants, and often co-surfactants with typical droplet sizes between 20 and 200 nm. Despite being thermodynamically unstable, nanoemulsion maintain high physical stability and long shelf life due to their small droplet size and optimized interfacial composition. They have emerged as effective vehicles for topical and transdermal drug delivery, enhancing drug solubility, dermal penetration, and bioavailability for both hydrophilic and lipophilic compounds, while also minimizing skin irritation [118,119,120].

In a 2019 study, Berlitz et al. [121] formulated a formulated an azelaic acid (AzA) nanoemulsion incorporating hyaluronic acid (HA) to improve cutaneous retention and therapeutic effect in the treatment of melasma and related hyper pigmentary disorders. The oil-in-water systems was prepared using rice bran oil, Span 80, Poloxamer 407, and ethanol with high-speed stirring.

The final formulation had a mean droplet size of 419 nm, zeta potential−10.9 mV, pH 5.01, and encapsulation efficiency of 84.65%, remaining stable over 30 days. TEM confirmed spherical morphology, while FTIR verified successful drug entrapment. In vitro skin permeation studies using porcine skin revealed significantly higher AzA retention with the HA-loaded nanoemulsion (14.35%) versus the HA-free formulation (8.66%) and conventional emulsion (6.77%). Additionally, tyrosinase inhibition was greater (IC_50_ = 13.97 mM) than both free AzA and arbutin. Cytocompatibility was confirmed, and sensory evaluation in volunteers showed better spreadability and reduced stickiness compared to commercial AzA cream [121].

In a 2024 study, Farooqui et al., developed a statistically optimized AzA nanoemulsion for enhanced skin delivery. The formulation, prepared by high-speed homogenization and probe sonication, used castor oil and vitamin E as the oil phase, Tween 80 and Transcutol P as surfactant/co-surfactant, and PVA with xanthan gum in the aqueous phase. Optimization via Box–Behnken design yielded a formulation with droplet size 184 nm, PDI 0.202, zeta potential −35.9 mV, and encapsulation efficiency 84.92%. Structural analyses (FTIR, DSC, TGA) confirmed drug stability and incorporation without chemical interaction. In vitro release showed a sustained zero-order release of 87.67% over 12 h (R^2^ = 0.9952), and ex vivo permeation studies demonstrated significantly enhanced skin penetration (81.2%) and retention (10.33 µg/cm^2^) compared to conventional emulsions. The formulation exhibited pseudoplastic flow, skin-compatible pH (5.62), and excellent stability under centrifugation, freeze–thaw cycles, and long-term storage at both 5 °C and 25 °C.

These results underscore the potential of nanoemulsion-based carriers for AzA, offering enhanced penetration, prolonged release, reduced irritancy, and superior patient acceptability in topical treatments for melasma, acne, and hyperpigmentation disorders [122].

### 5.3. Emerging Nanocarrier Systems for Potential Application in Azelaic Acid Delivery

Although several nanocarrier systems have been investigated for the topical delivery of AzA, numerous advanced platforms widely studied in dermatology remain largely unexplored for this specific compound. Emerging nanotechnologies, such as cubosomes, dendrimers, and polymeric nanoparticles, have demonstrated significant potential in enhancing skin penetration, drug stability and targeted delivery of other dermatological agents.

However, their application to AzA remains predominantly theoretical or in early-stage research. These systems warrant further investigation as they may offer innovative solutions to overcome the physicochemical limitations of AzA and expand its therapeutic utility in topical formulations.

#### 5.3.1. Cubosomes (CBs)

**Figure 10:**
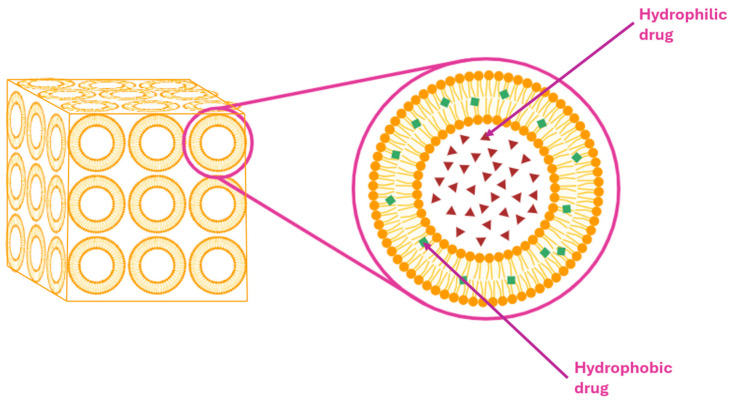
Cubosomes as nanostructured carriers with a bicontinuous cubic phase, enabling stable encapsulation and delivery of both hydrophilic and lipophilic drugs for topical applications.

Cubosomes (Figure 10) are nanostructured lipid-based drug carriers characterized by bicontinuous cubic-phase architecture, offering distinct advantages for topical drug delivery. These systems self-assembly into thermodynamically stable, three-dimensional lipid bilayer networks containing interpenetrating aqueous and lipid channels, thereby enabling the encapsulating of both hydrophilic and lipophilic drugs.

Their structural resemblance to the stratum corneum, along with intrinsic bioadhesive properties, enhances dermal compatibility, facilitate deeper penetration, and promotes prolonged drug retention in the epidermis.

Typical cubosome formulation employ glyceryl monooleate (GMO) and phytantriol, as the lipid phase, stabilized by surfactants such as Pluronic F127 to ensure colloidal stability. Drug release from cubosomes follows diffusion-controlled mechanisms and conforms to kinetic models such as Higuchi or Korsmeyer–Peppas, supporting sustained and controlled drug delivery. Compared to conventional vesicular systems, such as liposomes, ethosomes, and transfersomes, cubosomes demonstrate superior physical stability, enhanced skin drug deposition, and reduced drug leakage. Although AzA has not yet been formulated in cubosomes-based formulations, the unique properties of these carriers, including their ability to enhance solubility, mitigate local irritation, and improve skin retention, make them highly suitable candidates for future development in AzA topical delivery. Their tunable particle size, high drug-loading capacity, and favorable safety profile further underscore their potential in advancing dermatological therapies [123,124].

#### 5.3.2. Dendrimers

**Figure 11:**
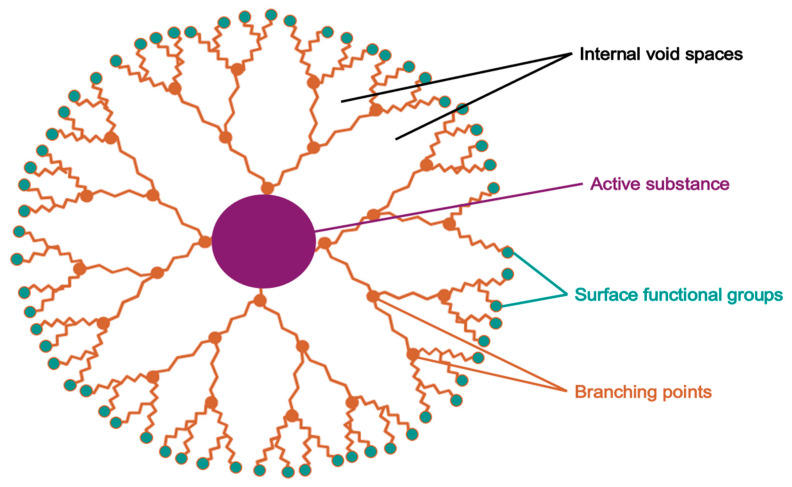
Dendrimers as highly branched macromolecules with internal cavities and functional surfaces, enabling targeted, controlled, and efficient topical drug delivery.

Dendrimers (Figure 11) are synthetic, highly branched, monodisperse macromolecules exhibiting a tree-like, three-dimensional architecture. They consist of a central core, multiple concentric layers (generations) of repeating monomer units, and a terminally functionalized surface. Their unique structure, along with the presence of internal cavities and multifunctional surface groups, enables dendrimers to encapsulate, conjugate, or adsorb active pharmaceutical ingredients, allowing for targeted and controlled drug delivery. Owing to their high drug-loading capacity, precise molecular dimensions, low polydispersity, and tunable surface chemistry, dendrimers have demonstrated considerable utility in enhancing drug solubility, permeability, and biocompatibility in topical formulations [125,126,127].

Table 4 poly(amidoamine) (PAMAM G4) dendrimers. The microsponges were prepared via a quasi-emulsion solvent diffusion, incorporating dithranol, G4 PAMAM dendrimers, and ethyl cellulose in the organic phase, and polyvinyl alcohol in the aqueous phase. Among five tested formulations, the optimized formulation (F3) showed an encapsulation efficiency of 62.3%, production yield of 66.3%, and a particle size of approximately 100 μm. Scanning electron microscopy confirmed spherical and porous structures, while FTIR and UV spectroscopy verified the absence of chemical interactions between dendrimer and drug.

The optimized microsponge system was incorporated into a Carbopol 934-based gel, exhibiting suitable pH (5.2–6.8) and spreadability for topical application. In vitro release studies demonstrated sustained drug release over 8 h, with GL-3 formulation achieving 70% cumulative release. Stability testing and in vivo dermal safety studies confirmed long-term physical integrity and non-irritant behavior.

Pharmacokinetic analysis revealed an extended-release profile and increased systemic exposure (AUC: 32 μg·h/mL) compared to conventional gels. These findings underscore the potential of dendrimer-based systems to improve topical delivery of poorly permeable drugs, such as AzA, by enhancing solubility, stability, and skin compatibility [128].

#### 5.3.3. Polymeric Nanoparticles

Polymeric nanoparticles (PNPs) are submicron-sized colloidal carriers, typically ranging from 10 to 1000 nm in diameter, and are synthesized from natural or synthetic polymers such as chitosan, ethyl cellulose, polycaprolactone, and polylactide-*co*-glycolide. These systems are widely employed to enhance drug stability, enable controlled and sustained release, and improve topical skin delivery by increasing drug residence time and modulating transdermal penetration.

Among the various polymers, chitosan has received particular attention due its favorable biocompatibility, mucoadhesive properties, and regulatory approval by the FDA for cutaneous use. Depending on their architecture, PNPs may function as nanospheres (matrix systems) or nanocapsules (reservoir systems), with drug release governed by mechanisms such as swelling, diffusion, enzymatic cleavage, and surface desorption. Several studies have demonstrated the efficiency of chitosan-based PNPs in dermal applications. For instance, nanoparticles loaded with alpha-arbutin or vitamin C, have shown enhanced skin deposition and tyrosinase inhibition than conventional formulations. Similarly, chitosan micelles loaded with glabridin (a prenylated isoflavan) showed improved skin permeation and suppression of melanogenesis. Given these promising results, polymeric nanoparticles represent a compelling strategy the topical delivery of AzA. Their ability to enhance the solubility of poorly water-soluble actives, reduce local irritation, and support sustained, localized drug release positions them as highly suitable carriers for addressing the formulation challenges associated with AzA and improving its therapeutic performance in dermatological applications [129,130].

#### 5.3.4. Transethosomal Gel Systems

Transethosomal gels are advanced topical drug delivery platforms that incorporate transethosomes-ultra-deformable lipid vesicles composed of phospholipids, ethanol, and edge activators-within a gel matrix, typically hydrogel-based. This combination enhances skin permeation, drug encapsulation, stability, and cutaneous residence time, making these systems particularly suitable for the delivery of poorly permeable or irritant-prone active pharmaceutical ingredients [131,132,133].

Nasr et al. [134] developed a transethosomal gel system to improve the skin delivery and antifungal activity of AzA. Using the thin-film hydration technique and 2^3^ full factorial experimental design, the authors optimized a formulation comprising soy lecithin, Labrafil M 1944 SC as the edge activator, and 40% ethanol. The resulting transethosomes exhibited a mean particle size of 219.8 ± 4.7 nm, a zeta potential of −36.5 ± 0.73 mV, and an entrapment efficiency of 81.9 ± 1.4%. Transmission electron microscopy confirmed the presence of spherical, non-aggregated vesicles, while FTIR spectroscopy verified successful drug encapsulation.

In vitro release studies demonstrated a sustained release of AzA from the gel (89% over 48 h) compared to 45% for the free drug. Ex vivo permeation studies using mouse skin revealed markedly enhanced dermal penetration (3056 µg/cm^2^ vs. 590 µg/cm^2^), attributed to ethanol-mediated stratum corneum disruption. The formulation also showed superior in vitro antifungal efficacy, with a MIC_90_ of 0.01% against *Trichophyton rubrum* compared to 0.32% for free AzA. In vivo, the transethosomal gel achieved an 83% cure rate in guinea pigs infected with *Microsporum canis* and *T. mentagrophytes*, outperforming both conventional AzA gel and oral itraconazole (66.76%). These findings suggest that transethosomal encapsulation significantly enhances the dermal delivery, bioactivity, and therapeutic potential of AzA, particularly in the treatment of superficial fungal infections such as dermatophytosis [135].

#### 5.3.5. Mesoporous Silica Nanoparticles (MSNs)

MSNs are inorganic nanocarriers characterized by their highly ordered, porous structure with pore diameters typically ranging from 2 to 50 nm. These nanoparticles exhibit a large surface area, tunable pore volume, and exceptional drug-loading capacity along with excellent biocompatibility. Their surfaces can be easily functionalized, enabling targeted drug delivery, stimuli-responsive release, and the co-delivery of multiple therapeutic agents [134,136].

To enhance skin delivery and anti-pigmentation efficacy, a mesoporous silica nanoparticle (MSN) gel formulation loaded with AzA was developed. MSNs were synthesized via the sol–gel method using CTAB and TEOS, followed by acid extraction and drug loading. The resulting AzA-MSNs exhibited a particle size of 211.9 nm, a zeta potential of −17.6 mV, and an entrapment efficiency of 93.46%, as measured by UV-spectrophotometry. FE-SEM analysis confirmed spherical, porous particles, while XRD and DSC confirmed the amorphous encapsulation of AzA within the MSN structure, contributing to its improved stability. FTIR spectroscopy verified successful drug loading and template removal. Nitrogen adsorption studies (BET) showed a pore size of 2.47 nm and surface area of 602.5 m^2^/g, indicating effective drug incorporation. The optimized MSN-AzA formulation demonstrated significantly improved tyrosinase inhibition and a cumulative drug permeation of 85.53% in ex vivo rat skin studies, compared to 43.15% from a control gel. Rheological analysis confirmed a pseudoplastic, non-Newtonian gel behavior, while 3-month stability studies across different temperatures confirmed physical and chemical integrity, with no liquefaction or drastic pH shifts. The findings underscore the potential of AzA-loaded MSNs for stable, effective topical pigmentation therapy, supporting their application in future dermatological treatments [137].

#### 5.3.6. Lyotropic Liquid Crystals (LLCs)

LLCs are self-assembled nanostructured systems formed by amphiphilic molecules (e.g., surfactants or lipids) in the presence of a solvent, typically water. These structures exhibit a unique combination of fluid-like behavior and long-range crystalline order. Depending on their concentration and composition, LLCs can organize into distinct internal geometries including lamellar, hexagonal, or cubic phases. Their biocompatibility, internal compartmentalization, and capacity for sustained release make them attractive platforms for the controlled drug delivery or poorly water-soluble or labile therapeutic agents [138,139].

In a study by Gowda et al. [140], LLC nanoparticles were developed using a bottom-up approach to enhance the topical delivery and therapeutic efficacy of AzA in the treatment of acne vulgaris. The formulation employed glyceryl monooleate, poloxamer 407, polyvinyl alcohol, and ethanol, and was optimized using a central composite design. The best-performing batch exhibited a particle size of 184.2 nm, a zeta potential of −16.1 mV, and an entrapment efficiency of 73.37%. Transmission electron microscopy confirmed spherical, well-dispersed vesicles, while ATR-FTIR and DSC analyses verified drug-excipient compatibility. The LLC formulation was incorporated into a Carbopol 934 gel for topical application. In vitro Drug Release studies revealed a biphasic release profile, with 91.07% cumulative release over 24 h—substantially outperforming a marketed AzA gel (59.88%). Ex vivo skin permeation studies demonstrated enhanced penetration (86.56%) and significantly higher cumulative retention in the skin (146.12 µg/cm^2^) compared to the commercial reference (58.58 µg/cm^2^). Acute dermal irritation testing confirmed the formulation’s safety profile. Moreover, the LLC gel exhibited strong antimicrobial activity against *Cutibacterium acnes*, *Staphylococcus epidermidis*, and *Staphylococcus aureus*, with inhibition zones comparable to or exceeding those of commercial products. These findings confirm the potential of LLC-based delivery systems to improve skin penetration, drug retention, and antimicrobial efficacy, highlighting their value in the topical management of acne vulgaris [141].

Table 5 presents an overview of the conventional and emerging nanocarrier systems already tested or proposed for the topical delivery of AzA, highlighting their main advantages and limitations.

### 5.4. Pharmaceutical Cocrystals: Crystal Engineering for Solubility and Delivery

Co-crystals are multi-component crystalline structures composed of an active pharmaceutical ingredient (API) and a co-former, both solid at room temperature, held together in a single crystal lattice via non-ionic interactions such as hydrogen bonding. Unlike salts or polymorphs, cocrystals preserve the neutral form of the API and are primarily employed to enhance solubility, bioavailability, stability, and mechanical properties of drugs without altering their pharmacological activity [140].

Zotova et al. [142] investigated innovative multicomponent solid and liquid drug phases by developing co-crystal and ionic liquid formulations of AzA and lidocaine (LID) through mechanochemical synthesis across various stoichiometric ratios.

Mixtures with LID molar fractions ranging from 0.1 to 0.9 yielded at least two novel crystalline phases and two new liquid forms. A key outcome was the formation of a 2:1 LID/AzA cocrystal, confirmed by single-crystal X-ray diffraction (SCXRD), featuring hydrogen bonding involving both AzA carboxyl groups and LID amine sites. Additionally, a novel 2:3 LID: AzA oligomeric ionic liquid phase was identified, representing the first reported example of such a structure involving dicarboxylic acid anions. Characterization via PXRD, DSC, FTIR, TGA, and NMR demonstrated that varying the LID/AzA ratio significantly influenced the thermal behavior, crystallinity, and ionicity of the formulations. Equimolar mixtures exhibited the highest degree of ionization and liquid formation, whereas higher AzA content favored ionic liquids with complex hydrogen bonding networks. These findings highlight AzA’s potential in non-traditional pharmaceutical formulations such as cocrystals and ionic liquids, enabling modulation of physicochemical properties and expanding its topical delivery applications [142].

In their 2022 study, Yarava et al. [143] explored the supramolecular architecture of a cocrystal composed of AzA and isonicotinamide (INIC) using advanced solid-state NMR (ssNMR) techniques supported by GIPAW-based quantum chemical calculations. The cocrystal, synthesized via a 1:1 molar grinding followed by methanol recrystallization, was structurally analyzed without requiring single crystals, highlighting the efficiency of ssNMR over traditional X-ray diffraction. Key NMR experiments—including 1D and 2D ^1^H-^13^C CP-MAS, ^1^H DQ-^1^H SQ correlation, and CP-reverse CP—enabled precise spectral assignments and the identification of hydrogen bonding patterns. The analysis revealed that the two OH protons of AzA exhibit distinct chemical shifts (13.6 and 16.2 ppm), indicative of different local environments, heterosynthon formation rather than symmetric acid dimers. Detailed DQ-SQ correlations confirmed the presence of both acid–amide and acid–pyridine heterosynthons as primary structural motifs within the cocrystal. These observations were corroborated by GIPAW-calculated chemical shifts, which closely matched experimental data. This work demonstrates that ssNMR, combined with computational modeling provides a powerful and efficient approach to elucidate structural features of pharmaceutical cocrystals, offering valuable insights for rational coformer selection and formulation design involving AzA [143].

Further experimental validation of these systems of AzA delivery is warranted.

### 5.5. Deep Eutectic Solvents

#### 5.5.1. Fundamentals and Pharmaceutical Classification

Recent advances in AzA delivery have increasingly focused on deep eutectic solvents (DESs), an emerging class of liquid systems comprising biocompatible and biodegradable components [22]. These systems serve multifunctional roles as solvents, permeation enhancers, and functional excipients. In 2023, DES formulations containing choline chloride, malonic acid, and polyethylene glycol (PEG-400) were shown to improve cutaneous diffusion of AzA, while maintaining formulation stability and exhibiting excellent tolerability [144].

Importantly, some DESs incorporate AzA as a structural component of the solvent matrix, forming therapeutic eutectic systems (THEDESs) that offer additional benefits including antimicrobial activity and rheological modulation. Such features position DESs as advanced multifunctional platforms in dermatological formulation science [22].

DESs are formed by combining a hydrogen bond acceptor (HBA), typically a quaternary ammonium or phosphonium salt, with a hydrogen bond donor (HBD), such as an organic acid, polyol, or amide. The extensive hydrogen bonding network between these components disrupts their individual crystalline lattices, resulting in a homogeneous liquid phase at temperatures significantly below the melting points of the pure constituents, a phenomenon known as deep melting point depression that distinguishes DESs from conventional solvents and liquid mixtures [144].

A classic example is the eutectic system composed of choline chloride (HBA) and urea (HBD) mixed at a 1:2 molar ratio, yielding a liquid at room temperature despite both starting materials being solids under ambient conditions [145]. Due to their shared physicochemical features such as negligible vapor pressure, tunable polarity, and high solubilizing capacity, DESs are often considered analogs of ionic liquids; however, they are generally simpler to synthesize and comprise less toxic, more environmentally friendly components [146].

**Figure 12:**
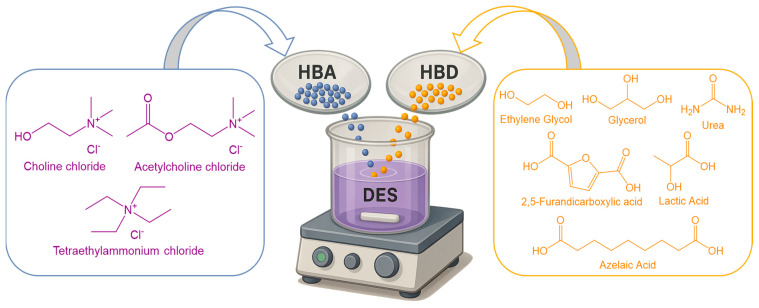
Examples of Type III DES possessing a HBA (quaternary ammonium salts) and a HBD (alcohols, carboxylic acids and amines).

DESs are typically categorized into four types (Table 6):

- Type I: Metal salt + metal salt (e.g., AlCl_3_ + NaCl);
- Type II: Metal salt + hydrated metal salt;
- Type III: Quaternary ammonium salt (e.g., choline chloride ChCl) + HBD (e.g., glycerol);
- Type IV: Metal salt + hydrogen bond donor;

Among these, Type III DESs (examples are shown in Figure 12) predominate in pharmaceutical and biomedical applications due to their favorable safety profiles and formulation versability. The vast array of potential HBA-HBD combinations enables fine-tuning of physicochemical properties such as viscosity, polarity, and solubilizing power, facilitating the design of bespoke solvents optimized for topical drug delivery [144,145,146,147,148].

#### 5.5.2. Physicochemical Properties of DESs

DESs exhibit unique physicochemical characteristics that render them particularly attractive as functional excipients in pharmaceutical formulations, with increasing application in topical drug delivery systems.

**Solvating capacity:** One of the most notable attributes of DESs is their exceptional solvating power, which arises from the dense hydrogen bonding network established between the hydrogen bond acceptor (HBA) and hydrogen bond donor (HBD) components. This extensive intermolecular interaction disrupts the crystalline lattice of solutes, facilitating dissolution of both hydrophilic and hydrophobic compounds at concentrations exceeding those achievable in aqueous media. For poorly soluble drugs such as AzA (AzA), DES-based formulations—particularly those classified as therapeutic deep eutectic solvents (THEDESs)—have demonstrated substantial solubility enhancement by enabling the drug to integrate molecularly into the eutectic matrix [22].

**Volatility and thermal stability:** DESs typically exhibit negligible vapor pressure, a property that minimizes minimizing solvent loss via evaporation and supporting prolonged stability in dermal applications. Although generally thermally stable under ambient or physiological temperatures, some DESs—especially those incorporating choline chloride—may undergo degradation above 80 °C, releasing volatile by-products such as trimethylamine and hydrochloric acid. Consequently, comprehensive thermal stability assessments under varied storage conditions are essential during formulation development [146,149].

**Rheological properties and viscosity**: DESs often possess relatively high viscosity at room temperature, attributed to their extensive hydrogen bonding and the bulky nature of constituent molecules Elevated viscosity can impede solute diffusion and affect drug release kinetics. However, incorporation of small amounts of biocompatible co-solvents, such as polyethylene glycol (PEG-400) or glycerol effectively reduces viscosity. Moderate viscosity is pharmaceutically beneficial for topical products, by enhancing skin residence time and enabling controlled drug release while preventing run-off [144,150].

**Polarity and tunability:** The polarity of DESs is highly tunable via selection of appropriate HBA-HBD combinations. Hydrophilic DESs (choline chloride/glycerol) are fully miscible with water and are suitable for polar drugs, whereas hydrophobic DESs (menthol/fatty acids) favor lipophilic active ingredients. This tunability facilitates optimization of partition coefficients between the formulation and the stratum corneum, critical for dermal drug delivery. For amphiphilic drugs like AzA, moderately polar DESs optimize solubilization and skin permeation [151].

**Biocompatibility and toxicological profile:** Most DESs are composed of naturally derived, food-grade, or pharmaceutically acceptable materials, including choline, amino acids, organic acids, and polyols, conferring favorable safety profiles. In vitro and in vivo studies indicate minimal cytotoxicity and skin irritation for a range of DES compositions. For example, Aza-formulations in choline chloride–malonic acid–PEG systems have demonstrated good dermal tolerability in both animal models and preliminary human studies. Nevertheless, each DES formulation requires individual toxicological evaluation to account for component-specific toxicity, impurity effects, and sensitization potential upon long-term or repeated use [22,144].

#### 5.5.3. DES in Pharmaceutical Drug Delivery: Solubility and Skin Penetration Enhancement

Deep eutectic solvents (DESs) have gained significant attention in pharmaceutical sciences due to their capacity to enhance active pharmaceutical ingredients (APIs) solubility and transdermal permeation. Their unique physicochemical milieu facilitates both molecular dissolution and the subsequent release of solubilized compounds into biological substrates such as skin [152,153].

#### 5.5.4. Solubility and Dissolution Enhancement by DES/THEDES

Many dermatological agents, including AzA, suffer from limited aqueous solubility, restricting topical bioavailability. DESs overcome this limitation by forming therapeutic deep eutectic solvents (THEDES), where the API often acts as hydrogen bond donor (HBD) within the eutectic system, producing a molecularity homogeneous liquid phase that circumvents crystalline dissolution and markedly enhances thermodynamic activity [22].

Empirical evidence supports this mechanism. Aroso et al. [154] demonstrated that THEDESs formed with choline chloride or menthol with organic acids achieved markedly increased dissolution rates compared to the pure crystalline forms. Similarly, eutectic systems composed of ibuprofen and thymol have shown a pronounced enhancement in solubility and dissolution kinetics.

#### 5.5.5. Enhancement of Skin Penetration and Drug Delivery

The stratum corneum forms the primary barrier to topical drug adsorption, due to its lipophilic nature, limiting diffusion of polar or large molecules. AzA, with moderate molecular weight of 188.22 g/mol and moderate polarity, exhibits low percutaneous absorption. Traditional penetration enhancers have limited efficacy; however, deep eutectic solvents (DESs) offers innovative dual-function systems that enhance solubility and dermal delivery [22,155]. DESs can enhance skin penetration via multiple mechanisms. Component like menthol and fatty acids disrupt stratum corneum lipids organization, increasing membrane fluidity and permeability. For instance, a menthol–lauric acid DES significantly enhanced diclofenac permeation through porcine skin by both solubilizing the drug in a high-energy state and disrupting lipid organization [22]. Moreover, DESs modulate drug thermodynamic activity. While strong solvation in polar DESs may inhibit drug release, adding co-solvents such as PEG-400 raises drug activity and promotes diffusion. Luhaibi et al. [144] demonstrated that PEG-containing DESs facilitated partitioning of AzA into a lipophilic phase, mimicking the stratum corneum, and enhanced skin delivery.

A notable DES/ionic liquid hybrid, choline geranate (CAGE), has shown enhanced delivery of small molecules and biomacromolecules Clinical studies using CAGE-based gels showed therapeutic efficacy in rosacea, highlighting DES potential as penetration enhancers with intrinsic pharmacological activity [39].

For AzA, DESs mixtures such as malonic acid/choline chloride/ have outperformed standard formulations in vitro diffusion assays. Although ex vivo skin data remain sparse, in vivo outcomes suggest improved delivery [144]. Analogously, risperidone-capric acid THEDESs exhibited markedly increased transdermal flux, reinforcing the relevance of fatty acid-based eutectics as dual-role agents [156].

Overall, DESs offer customizable platforms for skin delivery by modulating solubility, drug activity, barrier disruption, barrier disruption and skin hydration, making them highly adaptable for targeting diverse dermatological conditions.

#### 5.5.6. DES-Based Topical Formulations of Azelaic Acid and Related Systems

Recent research highlights DESs’ utility in topical AzA formulations. Luhaibi et al. [144], systematically evaluated various choline chloride–malonic acid DES systems, both binary and ternary (with PEG 400). The optimized formulation (1:1:6 molar ratio of ChCl:MA:PEG), termed MCP116, displayed a viscosity and spreading profile comparable to a commercial AzA gel significantly enhanced in vitro permeation, and maintained physicochemical stability over one month. Additionally, MCP116 demonstrated superior antimicrobial activity and skin tolerability, emphasizing its promise as a next-generation AzA topical vehicle [144].

Hung et al. [50] integrated eutectic principles into a microemulsion system for AzA increasing oil-phase solubility and bioavailability despite not constituting conventional DESs. This underscores eutectic concepts’ adaptability for formulation design beyond classical DES structures.

In parallel, Tomić et al. [157] explored AzA delivery via nanocrystal hydrogels, enhancing skin penetration through particle size reduction. Though distinct from DES technology, this strategy shares a common goal—overcoming AzA’s solubility limitations. Although this approach differs from deep eutectic solvent (DES) technology, both strategies share the common objective of overcoming the solubility limitations of AzA. Comparative analyses suggest that DESs may provide equal or superior dermal delivery by solubilizing AzA at the molecular level, while concurrently exerting synergistic antibacterial effects attributable to the acidic components of DES formulations.

Further studies on other pharmacological agents underscore the versatility of DESs. Al-Akayleh et al. [158,159] have demonstrated the effectiveness of therapeutic DESs (THEDES) for transdermal and wound healing applications, while Mitragotri et al. [160] utilized choline-based ionic liquids to deliver macromolecules like insulin across the skin—an achievement previously considered unfeasible.

Notably, Yang et al. [161] introduced the concept of eutectogels—polymer-based gels containing DESs—as an innovative platform for microneedle-assisted acne therapy. These systems synergically combine physical and chemical enhancement mechanisms, offering targeted, high-efficiency drug deposition into the skin. Although AzA was not evaluated in this context, its incorporation into analogous eutectogel systems appears both feasible and promising. Collectively, these case studies reinforce DESs’ multifaceted utility in enhancing drug solubility, skin permeation, and therapeutic efficacy. They offer a design framework that can be adapted to AzA and similar actives, supporting the development of safer, more effective, and innovative dermatological treatments.

### 5.6. Quality-by-Design (QbD) Optimization

The Quality-by-Design (QbD) approach that integrates risk assessment with statistically designed experimentation, has become a recurrent framework for developing topical azelaic-acid (AzA) dosage forms [105,162]. In a central-composite design study, Mistry & Ravikumar optimized the composition of a ethosomal AzA gel by carrying phospholipid, cholesterol and ethanol concentrations.

The optimized vesicles (~4.3 µm, ~92% entrapment) increased ex vivo pig-skin permeation thought pig skin and antibacterial activity compared to a carbopol gel and a marketed 20% AzA cream, while remaining physiochemically stable for three months under ICH conditions [105]. Similarly, Salimi et al. [162] employed a fractional factorial design using Minitab software to optimize surfactant/co-surfactant/water ratios in an oil-in-water microemulsion system. The resulting nanosized droplets (48–65 nm) significantly increased AzA flux (*p* < 0.05) across both hairy and non-hairy guinea pig skin relative to an aqueous AzA solution. Moreover, the formulation exhibited stability against phase separation and drug degradation during six months of stress testing.

### 5.7. Challenges and Considerations on Integration of Artificial Intelligence in Azelaic Acid Therapy

The integration of artificial intelligence (AI) tools into AzA therapy faces challenges common to dermatology, including algorithmic bias, data privacy issues, and difficulties in clinical workflow integration [163]. Effective AI model training requires large, high-quality datasets; however, dermatological endpoints often depend on subjective clinician or patient evaluations, which introduce variability and reduce model generalizability [164,165]. Because erroneous automated recommendations could compromise care, every AI-generated output must undergo human verification, and current clinical-decision-support guidance stresses multidisciplinary oversight, validation and post-deployment monitoring to safeguard patient safety [166,167]. From a formulation standpoint, advanced carriers such as liposomes, solid-lipid or polymeric nanoparticles add manufacturing steps, specialized equipment and stringent quality-control requirements, factors that drive up cost and complicate large-scale production [168,169]. Consistent batch reproducibility, solvent removal and sterility assurance are frequently identified as critical bottlenecks, which can be mitigated—but not eliminated—by continuous-flow methods and other Quality-by-Design (QbD) strategies that map critical process parameters and automate real-time release testing [170,171].

Consequently, the broad adoption of AI-guided, nanotechnology-enabled AzA therapies will hinge on parallel advances in curated dermatology datasets, clinician-centered oversight frameworks, and QbD-driven scale-up solutions that lower unit cost while preserving product quality [172].

Regulatory considerations remain a significant barrier. Any novel AzA vehicle—whether a nanoparticle gel or an AI-driven decision-support platform—must satisfy the full FDA/EMA approval or clearance pathway, the same evidentiary standards that already challenge nanomedicine products. The FDA reports a year-on-year rise in drug-application dossiers that contain artificial-intelligence (AI) components, signaling agency openness to data-driven tools across the product life-cycle [173].

Dermatology is particularly well suited for personalized approach, as diagnosis and treatment responses are heavily image-based and are already being augmented by AI algorithms that individualize cosmetic and therapeutic plans [174,175,176]. The convergence of advanced formulation science and AI-guided personalization is therefore poised to expand AzA’s therapeutic index, delivering higher efficacy across diverse indications while minimizing irritation through precision dosing and targeted skin delivery.

## 6. Combination Therapies

### 6.1. Synergistic Combinations (Azelaic Acid with Retinoids, Antibiotics, Antioxidants)

AzA is frequently used in combination with other therapeutic agents to enhance efficacy various dermatological conditions. Synergistic regimens targeting multiple pathogenic pathways have shown promise in treating acne, rosacea, and hyperpigmentation. Evidence from randomized controlled trials demonstrates added benefit when AzA is used with topical clindamycin [177] or with oral doxycycline [178], and with topical retinoids [179] in melasma.

### 6.2. Enhanced Dermatologic Outcomes with Azelaic Acid-Retinoid Combinations

Combining AzA with topical retinoids may yield additive or synergistic effects, especially in pigmentary disorders. For example, in melasma treatment, 20% AzA cream combined with 0.05% tretinoin produced faster and more pronounced pigment reduction than AzA alone [179]. In a 6–month controlled trial involving 50 patients, the combination led to a more rapid decline in MASI scores at 3 months and a higher proportion of “excellent” responders at study conclusion compared to AzA monotherapy [179]. Retinoids promote epidermal turnover and melanin dispersion [180], which may enhance AzA penetration and amplify its depigmenting effect [181].

Despite only moderate objective efficacy, irritation remains a major drawback. In a 12–week trial in Bangladesh evaluating the same combination, 40% of participants reported burning, and 20% developed erythema [182].

Independent studies on tretinoin monotherapy report erythema and desquamation in up to 88% of users, emphasizing the importance of gradual titration [183]. As triple-combination therapy (hydroquinone 4% + tretinoin 0.05% + fluocinolone acetonide 0.01%) consistently outperforms dual-agent regimens in meta-analyses, the AzA-tretinoin pairing may be best positioned as a hydroquinone-free alternative rather than a first-line option for severe melasma [184]. Evidence supporting the AzA-tretinoin combination in acne is limited. One case series involving four patients with truncal acne reported that nightly tretinoin 0.05% lotion combined with twice-daily AzA 15% foam led to clearance of inflammatory lesions and post-inflammatory hyperpigmentation without inducing new irritation [185].

### 6.3. Azelaic Acid and Antibiotics: A Dual Approach to Inflammatory Skin Disorders

AzA possesses bacteriostatic, keratolytic, and free radical-scavenging properties, contributing to both antimicrobial and anti-inflammatory effects [3]. These characteristics allow AzA to complement systemic antibiotics in acne management. In a randomized, open-label trial by Gollnick et al., 85 patients with nodular-papulopustular or conglobate acne were treated for six months with topical AzA 20% cream plus oral minocycline (100 mg/day), or standard-dose oral isotretinoin. The combination reduced papules and pustules by a median of 88%, compared to 97% with isotretinoin. It also completely cleared deep nodules but was associated with significantly fewer local (36.5% vs. 65.7%) and systemic (8% vs. 14.3%) adverse events, while avoiding isotretinoin’s teratogenicity. Approximately half of the patients in the combination group maintained remission during a subsequent 3-month AzA-only maintenance phase [186].

In papulopustular rosacea, a two-phase multicenter study enrolled 172 subjects who received open-label AzA 15% gel twice daily plus doxycycline 100 mg/day for up to 12 weeks. Mean inflammatory lesion reduction exceeded 70%. Responders were then randomized to receive AzA or vehicle for an additional 24 weeks: AzA maintained remission in 75% of patients and significantly delayed relapse [178]. Across these studies, oral tetracycline-class antibiotics were limited to 3–6 months in accordance with antimicrobial stewardship guidelines. The non-antibiotic nature of AzA enables its long-term use without promoting bacterial resistance [187].

## 7. Conclusions and Future Directions

This review set out to provide a comprehensive and critical overview of AzA (AzA) as a multifunctional dermatologic agent, with a particular emphasis on its formulation challenges, novel delivery strategies, and potential structural innovations to enhance its bioavailability and therapeutic performance. Special attention was given to the persistent limitations in AzA’s aqueous solubility, skin penetration, and compatibility with high-concentration topical formulations, which have long hindered its full clinical potential.

Recent advances in nanotechnology-based formulation approaches have emerged as the most promising research areas to overcome these limitations. Systems such as liposomes, ethosomes, niosomes, solid lipid nanoparticles (SLNs), nanosponges, nanoemulsions, transethosomes, mesoporous silica nanoparticles (MSNs), and lyotropic liquid crystals (LLCs) have shown considerable efficacy in improving AzA’s dermal absorption, retention, and tolerability, even at reduced concentrations. These technologies enable controlled and sustained release while mitigating local irritation, a key factor for chronic dermatologic conditions like acne, rosacea, and hyperpigmentation disorders.

Among emerging strategies, deep eutectic solvents (DESs) have demonstrated notable capacity to improve both solubility and biocompatibility of AzA, offering a unique solvent platform for stable, non-irritant, high-loading formulations. Additionally, cyclodextrin-based nanosponges significantly enhance AzA’s solubility and antimicrobial efficacy, further supporting their role in high-performance topical systems. Transethosomal gels, MSNs, and nanoemulsions with hyaluronic acid or vitamin E have further expanded the landscape of AzA-compatible excipient systems capable of enhancing both formulation esthetics and clinical outcomes.

In terms of future directions, structural modification strategies are gaining traction as a parallel path to improve AzA’s systemic bioavailability and formulation flexibility. One such approach is the development of pharmaceutical cocrystals, as exemplified by recent studies on AzA-lidocaine cocrystals and ionic liquids. These multicomponent systems offer enhanced dissolution profiles, improved thermal and mechanical stability, and better compatibility with other formulation agents, without altering AzA’s pharmacological activity. Moreover, biodegradable polymer-based nanocarriers and functionalized mesoporous structures provide new avenues for targeted delivery, stimuli-responsive release, and dose reduction, paving the way for advanced therapeutic systems with minimized adverse effects.

While the majority of research has centered on topical delivery, future investigations may extend to transdermal systems, microneedle-assisted administration, or dual-action formulations that combine AzA with synergistic agents (e.g., antioxidants, retinoids, anti-inflammatories) in a single nanosystem. Integration of AI-assisted formulation design, predictive skin permeation modeling, and smart carriers responsive to pH or oxidative stress could further refine the delivery and personalization of AzA-based therapies.

In conclusion, the path forward for AzA lies in the strategic integration of advanced formulation technologies with rational structural design, aiming to unlock its full potential as a safe, effective, and patient-friendly agent in both dermatologic and cosmetic applications. Continued interdisciplinary collaboration among pharmaceutical scientists, material chemists, and clinical dermatologists will be essential to bring these innovations from the bench to bedside.

## Figures and Tables

**Table 1:** Overview of synthetic routes for azelaic acid production.

Route	Starting Material	Key Reagent/Catalyst	Yield (%)	Notes	Ref.
Ozonolysis (classical)	Oleic acid	O_3_ + oxidant	70–95	Industrial route, concerns regarding ozone safety	[10]
H_2_O_2_-based oxidation	Vegetable oils	H_2_O_2_, catalysts	≤80	Industrialized by Matrica S.p.A. (Italy)	[10]
Performic acid + O_3_	Oleic acid	HCOOH + H_2_O_2_	up to 95	In situ peracid formation enhances selectivity	[11]
Biocatalytic	Oleic acid	Multi-enzyme cascade	5–10 mM	Low yield, multistep enzymatic route	[12]
Chemo-enzymatic	Oleic acid	Lipase + Fe(NO_3_)_3_ + TEMPO	44	No chromatography required	[12]
Fermentative	Oleic acid	*C. tropicalis* + ozonolysis	67	Long fermentation, no pelargonic acid	[12]
Chemical	Cyclohexanone + methyl acrylate	Percarboxylic acid + Pd catalyst	>90	Fully continuous processing	[13,14]

**Table 2:** Overview of the main biological activities of AzA which induce therapeutic effect.

Mechanism	Target	Therapeutic Effect	Ref.
Antimicrobial	*Cutibacterium acnes*, oxidoreductase	Inhibits bacterial growth and colonization	[41,42]
Anti-inflammatory	IL-1β, TNF-α, NF-κB, MMPs, LL-37	Suppresses inflammatory cytokines and oxidative stress	[43,44,45]
Antimelanogenic	Tyrosinase, hyperactive melanocytes	Inhibits melanin synthesis and melanocyte proliferation	[3,15,19,46,47]
Antioxidant	ROS, lipid peroxidation	Protects skin barrier and reduces oxidative tissue damage	[1,42,48]

**Table 3:** Mechanisms of action and biological targets of azelaic acid in dermatological applications.

Activity	Target/Mechanism	Effect/Result	Ref.
Antibacterial	Mitochondrial oxidoreductase inhibition	Impaired bacterial respiration and energy production leading to bacterial growth inhibition.	[5]
Antibacterial	KLK5 cleaves hCAP18 into LL-37	Increased LL-37 promotes inflammation in rosacea	[65]
Anti-inflammatory	IL-1β, TNF-α downregulation (via NF-κB)	Reduction in pro-inflammatory cytokine levels	[56]
Anti-inflammatory	MMP-9 inhibition	Inhibition of MMP-9 activity in inflamed skin	[56]
Antimelanogenic	Tyrosinase inhibition	Significant reduction in melanin index	[59]
Antimelanogenic	Reduced melanocyte proliferation	Decrease in melanocyte density on histological analysis	[58]
Antioxidant	Reactive oxygen species scavenging	Reduction in reactive oxygen species markers	[64]
Immunomodulatory	Suppression of pro-inflammatory cytokine milieu via PPARγ pathway	Modulation of cytokine balance in keratinocyte culture	[43]
Anti-keratinization	Inhibition of keratinocyte hyperproliferation via mitochondrial swelling and ER dilation	Reduction in hyperkeratosis and normalization of intra- and interfollicular keratinization	[3]
Microbiome modulation	Inhibition of quorum sensing signals in *C. acnes*	Reduction in pathogenic bacterial communication	[56]
Antiangiogenic	Inhibition of VEGF expression via PI3K/AKT pathway	Reduction in angiogenic factors in psoriatic skin	[45]
Photoprotective	ROS scavenging by plant polyphenols; enhancement of UV absorber stability	Reduction in UV-induced oxidative stress and potential mitigation of photodamage in sunscreen formulations	[66]

**Table 4:** Efficacy comparisons between azelaic acid and comparator treatments.

Condition	Formulation	Comparator	Duration	Efficacy Result	Ref.
Hyperpigmentation	Azelaic acid 20% cream	Hydroquinone 2%	24 weeks	Comparable efficacy; mild local irritation with azelaic acid	[79]
Hyperpigmentation	20% cream	Hydroquinone 4%	8–24 weeks	Greater MASI reduction with azelaic acid; adverse events similar between groups	[80]
Acne vulgaris	Azelaic acid chemical peel	Pyruvic acid peel	12 weeks	Both effective; azelaic acid better tolerated; pyruvic acid reduced sebum more	[81]
Rosacea	Azelaic acid 15% gel	Metronidazole 0.75% gel	15 weeks	Greater reduction in lesions and erythema with azelaic acid	[18]
Rosacea	Azelaic acid 20% cream	Dapsone 7.5% gel	12 weeks	Similar efficacy; azelaic acid had more local side effects; dapsone better tolerated	[82]
Rosacea	Azelaic acid 15% gel	Ivermectin, metronidazole, minocycline	12–16 weeks	Ivermectin is most effective; azelaic acid better than metronidazole; all well tolerated, with more irritation from azelaic acid	[83]
Acne	Azelaic acid 20% cream	Clindamycin 1% lotion	8 weeks	Similar efficacy; azelaic acid well tolerated; no direct tolerability comparison reported	[84]
Acne	Azelaic acid 15% gel	Adapalene 0.1% gel	12 weeks	Comparable efficacy; adapalene slightly better tolerated	[17]

**Table 5:** Conventional and emerging nanocarrier systems with azelaic acid.

Classical Nanocarriers Tested with Azelaic Acid						
Nanocarrier System	Status with AzA	Key Composition/Type	Main Findings/Notes	Advantages	Limitations	Ref.
Liposomes	Tested	Phospholipids (e.g., Phospholipon 50 IP)	Increased SC retention (187.5 mg/cm^2^), good stability, enhanced antimicrobial effects	Biocompatible, non-irritant	Risk of leakage, low long-term stability	[24,100]
Ethosomes	Tested	Phospholipids + high ethanol (20–45%)	Enhanced permeation, improved antimicrobial activity, sustained release (93.4% in 12 h)	High penetration, stable, effective	Potential irritation from ethanol	[34,105]
Niosomes	Tested	Non-ionic surfactants (Span 40), cholesterol	High encapsulation efficiency (72.3%), sustained release (82.7%), reduced irritation (with aloe vera)	Stable, biocompatible	Less studied than liposomes	[102]
SLNs	Tested	Solid lipids (stearic acid) + surfactants	Enhanced release (93.9%), reduced irritation (HET-CAM), improved depigmentation	Good stability, GRAS lipids	Size growth at room temp	[35]
NLCs	Proposed	Solid + liquid lipids	Literature-based evidence, ↑ skin penetration	Literature-based evidence, improved skin penetration	No specific AzA data reported	[110]
Nanosponges	Tested	β-Cyclodextrin crosslinked networks	Increased solubility (5-fold), better MIC/MBC than free AzA	Stable, non-irritant, good control	↓ Tyrosinase inhibition vs. free AzA	[37]
Nanoemulsions	Tested	O/W emulsions w/surfactants and HA or vitamins	Enhanced retention, greater tyrosinase inhibition, improved sensorial profile	Stable, well tolerated	Thermodynamically unstable	[121,122]
**Non-Classical/Emerging Nanocarriers Tested or Proposed with Azelaic Acid**						
**Nanocarrier System**	**Status with AzA**	**Key Composition/Type**	**Main Findings/Notes**	**Advantages**	**Limitations**	**Ref.**
Cubosomes	Not yet tested	GMO/phytantriol + Pluronic F127	Proposed: stable, SC-mimicking, dual drug encapsulation	Excellent skin compatibility	Theoretical only	[123,124]
Dendrimers	Early-stage	PAMAM G4 + microsponges (with dithranol)	↑ Stability, ↑ AUC, ↓ irritation (model drug)	Controlled release, high loading	No direct AzA formulation yet	[128]
Polymeric NPs	Suggested	Chitosan, PLGA, ethyl cellulose	Based on vit. C, arbutin, glabridin-potential for AzA	Mucoadhesive, biocompatible	Lacking direct AzA evidence	[129,130]
Transethosomes	Tested	Lecithin + ethanol + edge activator	↑ Permeation (3056 µg/cm^2^), ↑ antifungal activity, 83% cure rate	Deformable, high EE	Needs ethanol tuning	[135]
MSNs	Tested	Silica (CTAB/TEOS), high surface area	↑ Tyrosinase inhibition, ↑ permeation (85.5%), ↑ stability	Porous, functionalizable	Inorganic, limited biodegradability	[136]
LLCs	Tested	GMO + poloxamer + EtOH	↑ Release (91%), ↑ skin retention (146 µg/cm^2^), antimicrobial	Biphasic release, safe	Complex formulation, rheology control	[139]

**Table 6:** Types of Deep Eutectic Solvents [144,147].

Type	Formula	Terms	Example
I	M^+^X^−^ + z MCl_X_	M = Zn, Sn, Al, Ga, Fe, In	ZnCl_2_ + ChCl
II	M^+^X^−^ + z MCl_X_·Yh_2_O	M = Co, Cu, Ni, Fe, Cr	CoCl_2_·6H_2_O + ChCl
III	Cat^+^X^−^ + zRZ	RZ = OH, COOH, CONH_2_ (hydrogen bond donor)	Urea + ChCl
IV	MCl_X_ + RZ → MCl_X−1_^−^·RZ + MCl_x+1_^−^	M = Zn, Al and Z = OH, CONH_2_	ZnCl_2_ + urea

## Data Availability

Not applicable.
